# Cytotoxic necrotizing factor 1 hinders colon tumorigenesis induced by colibactin-producing *Escherichia coli* in *Apc^Min/+^* mice

**DOI:** 10.1080/19490976.2023.2229569

**Published:** 2023-07-07

**Authors:** Héloïse Chat, Guillaume Dalmasso, Catherine Godfraind, Virginie Bonnin, Racha Beyrouthy, Mathilde Bonnet, Nicolas Barnich, Amel Mettouchi, Emmanuel Lemichez, Richard Bonnet, Julien Delmas

**Affiliations:** a Centre de Recherche en Nutrition Humaine Auvergne, University Clermont Auvergne, Inserm U1071, INRAE USC 1382, Microbes, Intestin, Inflammation et Susceptibilité de l’Hôte (M2iSH), Clermont-Ferrand, France; b Neuropathology Unit, University Hospital of Clermont-Ferrand, Clermont-Ferrand, France; c Institut Universitaire de Technologie, University Clermont Auvergne, Clermont-Ferrand, France; d Institut Pasteur, University of Paris, CNRS UMR2001, Paris, France; e Department of Bacteriology, University Hospital of Clermont-Ferrand, Clermont-Ferrand, France

**Keywords:** Colorectal cancer; *Escherichia coli*; colibactin; CNF-1; CoPEC

## Abstract

Colorectal cancer (CRC) patients are frequently colonized by colibactin-producing *Escherichia coli* (CoPEC) (>40%), which enhances tumorigenesis in mouse models of CRC. We observed that 50% of CoPEC also contains the *cnf1* gene, which encodes cytotoxic necrotizing factor-1 (CNF1), an enhancer of the eukaryotic cell cycle. The impact of its co-occurrence with colibactin (Clb) has not yet been investigated. We evaluated the impact of CNF1 on colorectal tumorigenesis using human colonic epithelial HT-29 cells and CRC-susceptible *Apc*^*Min/+*^ mice inoculated with the CoPEC 21F8 clinical strain (Clb+Cnf+) or 21F8 isogenic mutants (Clb+Cnf-, Clb-Cnf+ and Clb-Cnf-). Infection with the Clb+Cnf- strain induced higher levels of inflammatory cytokines and senescence markers both *in vitro* and *in vivo* compared to those induced by infection with the Clb+Cnf+ strain. In contrast, the Clb+Cnf- and Clb+Cnf+ strains generated similar levels of DNA damage in HT-29 cells and in colonic murine tissues. Furthermore, the *Apc*^*Min/+*^ mice inoculated with the Clb+Cnf- strain developed significantly more tumors than the mice inoculated with the Clb+Cnf+ strain or the isogenic mutants, and the composition of their microbiota was changed. Finally, rectal administration of the CNF1 protein in *Apc*^*Min/+*^ mice inoculated with the Clb+Cnf- strain significantly decreased tumorigenesis and inflammation. Overall, this study provides evidence that CNF1 decreases the carcinogenic effects of CoPEC in *Apc*^*Min/+*^ mice by decreasing CoPEC-induced cellular senescence and inflammation.

## Introduction

Colorectal cancer (CRC) is the third most common cancer in the world, causing significant morbidity and mortality.^1^ CRC is a multifactorial disease involving both genetic and environmental factors. Among the genomic changes associated with CRC, loss-of-function mutations in the *Apc* (adenomatous polyposis coli) gene are the most prevalent and are considered the initiating event in approximately 80% of CRC cases.^2^ Among the environmental factors linked to CRC, the gut microbiota is increasingly thought to be a key player in CRC pathogenesis.^3,4^ Modification of the composition of the gut microbiota, or dysbiosis, has been reported in patients with CRC, with an increase in the abundance of bacteria such as *Bacteroides fragilis* or *Fusobacterium nucleatum* and a decrease in the abundance of *Faecalibacterium prausnitzii* .^5–9^

The involvement of the gut microbiota in CRC has been established using murine models of CRC. Germ-free *Apc*^*Min/+*^ mice display a lower number of intestinal and colorectal tumors than microbiota-bearing *Apc*^*Min/+*^ mice.^10^ A recent study showed that germ-free mice that received fecal samples from patients with CRC exhibited an increase in number of polyps, intestinal dysplasia, and levels of cellular proliferation markers as well as inflammation compared with those of germ-free mice that received fecal samples from healthy individuals.^11^

At the taxonomic level, analysis of the human CRC microbiome has identified potential microbial candidates implicated in CRC pathology, including *Escherichia coli*, *F. nucleatum*, and enterotoxigenic *B. fragilis* (ETBF).^8^ ETBF induced chronic inflammation and tumorigenesis in *Apc*^*Min/+*^ mice and led to high levels of interleukin-17 (IL-17) production, which disrupted normal myelopoiesis and resulted in the accumulation of pro-carcinogenic myeloid-derived suppressor cells in the tumor microenvironment.^12^ In *Apc*^*Min/+*^ mice, *F. nucleatum* increased tumor development without inducing colitis, accompanied by increased infiltration of myeloid cells into tumors.^7^

Recent studies have shown that pathogenic *E. coli* synthesizes toxins known as cyclomodulins, such as cytolethal distending toxins, cytotoxic necrotizing factor-1 (CNF1), cycle-inhibiting factor, and colibactin, which interfere with the cell cycle.^13,14^ Cyclomodulin-encoding genes, especially the colibactin-encoding *pks* island and CNF1-encoding gene (*cnf1*), are overrepresented in CRC patients colonized by *E. coli* strains.^15,16^

Colibactin-producing *E. coli* (CoPEC) strains have been identified in the colonic mucosa of approximately 55–67% of patients with CRC *versus* 19–21% of control patients.^15,16^ CoPEC has been shown to induce DNA double-strand breaks (DSB), chromosomal instability, genomic mutations and cell cycle arrest.^17–21^ CoPEC induces senescence of infected cells, accompanied by secretion of inflammatory mediators and growth factors, thus promoting proliferation of adjacent uninfected cells.^22^ Importantly, CoPEC promotes colon tumorigenesis in multiple murine models of CRC, including *Apc*^*Min/+*^ mice, AOM-treated *Il-10*^*–/–*^ mice, AOM/DSS-treated mice and *Apc*^*Min/+*^;*Il-10*^*–/–*^ mice.^16,22–24^ Notably, inflammation enhances the development of colon cancer in the *Apc*^*Min/+*^ model, which was established by specifically deleting the *APC* gene in epithelial cells,^25^ as seen with the use of dextran sulfate sodium (DSS)^26^ and by genetically introducing defective IL-10 signaling.^27,28^

The prevalence of *cnf1*-harboring *E. coli* is significantly higher in patients with CRC (37%) than in control patients (13%).^15^ Nevertheless, the involvement of *cnf1*-harboring *E. coli* in CRC has not been determined. CNF1 is a 115 kDa protein toxin that activates Rho GTPases, leading to cytoskeletal and cell cycle alterations with subsequent macropinocytosis and the formation of megalocytic, multinucleated cells.^29^ In addition, CNF1-induced activation of Rho GTPases triggers cellular events not directly linked to the actin cytoskeleton, such as the activation of NF-κB^30^ and the production of cytokines, such as IL-6 and IL-8,^31,32^ and provides protection against apoptosis.^33,34^ It also promotes quiescent cell entry into the cell cycle.^35^ Recently, Fabbri *et al*. showed, *in vitro*, that CNF1 induces epithelial-to-mesenchymal transition (EMT), a crucial step in malignant tumor conversion and invasiveness, in intestinal epithelial cells.^36^ A separate study has shown that CNF1 promotes the migration and invasion of prostate cancer cells *in vitro*.^37^ Therefore, it appears that many of the cellular activities induced by CNF1 might participate in carcinogenesis. Despite the overrepresentation of the *cnf1* gene in *E. coli* strains isolated from patients with CRC, the effect of the CNF1 toxin has not been studied in CRC.

In this study, we determined the prevalence of *E. coli* harboring the *pks* and *cnf1* genes in CRC patients and investigated the tumorigenic properties of *E. coli* strain 21F8 isolated from a human colon cancer biopsy and producing both colibactin and CNF1 in comparison with those of isogenic mutants using human intestinal epithelial HT-29 cells and an *Apc*^*Min/+*^ mouse model of CRC.

## Results

### Most E.coli strains harboring the cnf1 gene possess a pks island

The patient data used come from previous studies.^23,38^ The prevalence of *pks* was significantly higher in CRC patients (46%, *n* = 37/80) than in patients with diverticulosis (21%, *n* = 6/28; *p = 0.037*) (Table 1), which is in accordance with previously reported data.^15,16^ In contrast, the difference in the prevalence of *cnf1* in CRC patients (25%, *n* = 20/80) and diverticulosis patients (14%, *n* = 4/28; *p = 0.363*) was not significant. The majority of *E. coli* strains harboring the *cnf1* gene also carried the *pks* island: 95% (*n* = 19/20) and 100% (*n* = 4/4), in CRC patients and in healthy patients respectively, showing a strong association between the *cnf1* gene and the *pks* genomic island.

**Table 1.** Distribution of *E. coli* harboring *cnf1* and/or the *pks* genomic island producing colibactin (Clb) among CRC and control patients (percent in brackets).

Disease	Patients^3^	Clb+Cnf+	Clb+Cnf-	Clb-Cnf+	Clb-Cnf-^4^
CRC^1^	80	19 (23.8)	18 (22.5)	1 (1.3)	42 (52.5)
Control^2^	28	4 (12.0)	2 (8.0)	0 (0)	22 (80.0)

^1^CRC = patients with colorectal cancer.

^2^Control = patients with diverticulosis.

^3^Number of patients carrying a mucosa-associated E. coli that produces cyclomodulins (colibactin or CNF1).

^4^Number of patients carrying a mucosa-associated E. coli that does not produce cyclomodulins (colibactin or CNF1).

### Analysis of the virulome of CoPEC strains 11G5 and 21F8

We selected two CoPEC strains, 11G5 and 21F8, from CRC patients. Previous studies have reported that the 11G5 reference strain, which harbors only the *pks* island, increased the number of tumors in mice.^22,23,39^ The 21F8 strain possesses both *pks* and *cnf1*. We analyzed the virulome of these two CoPEC strains (11G5 and 21F8). Both of them belong to phylogenetic Group B2 and share 130 genes associated with virulence (Supplemental Figure S1). *In silico* analysis revealed major virulence factors belonging to the following five categories: adherence (type 1 fimbriae, FC1/S fimbriae, YadA fimbriae, and curli fiber), toxins (colibactin and Vat), iron uptake (enterobactin, ChuA, Sit, and yersiniabactin), protectin (*iss*), and motility and chemotaxis (*che, flg, fliA*, and *flh*). Virulence genes missing in 21F8 but present in the 11G5 genome were those involved in glutathionylspermidine amidase activity (*gsp* genes), resistance to mercury (*mer* genes), adhesion (*ehaB* and *espI*), invasion (*ibeA*) and iron uptake (*iro* genes). Virulence genes missing in 11G5 but present in the 21F8 genome were *pap* genes, which encode P fimbriae, adhesin genes (*iha, upaG*) and the *pic* gene, which encodes a colonization factor. Likewise, pathogenicity island II (PAI II), which harbors the *hlyCABD* operon and the *cnf1* gene, was present in *E. coli* strain 21F8 but absent in strain 11G5. Thus, the only cyclomodulin present in the 21F8 strain and absent in the 11G5 strain was the CNF1 toxin.

### The E.coli 21F8 strain lacking cnf1 promotes colonic tumorigenesis

**Figure 1.**
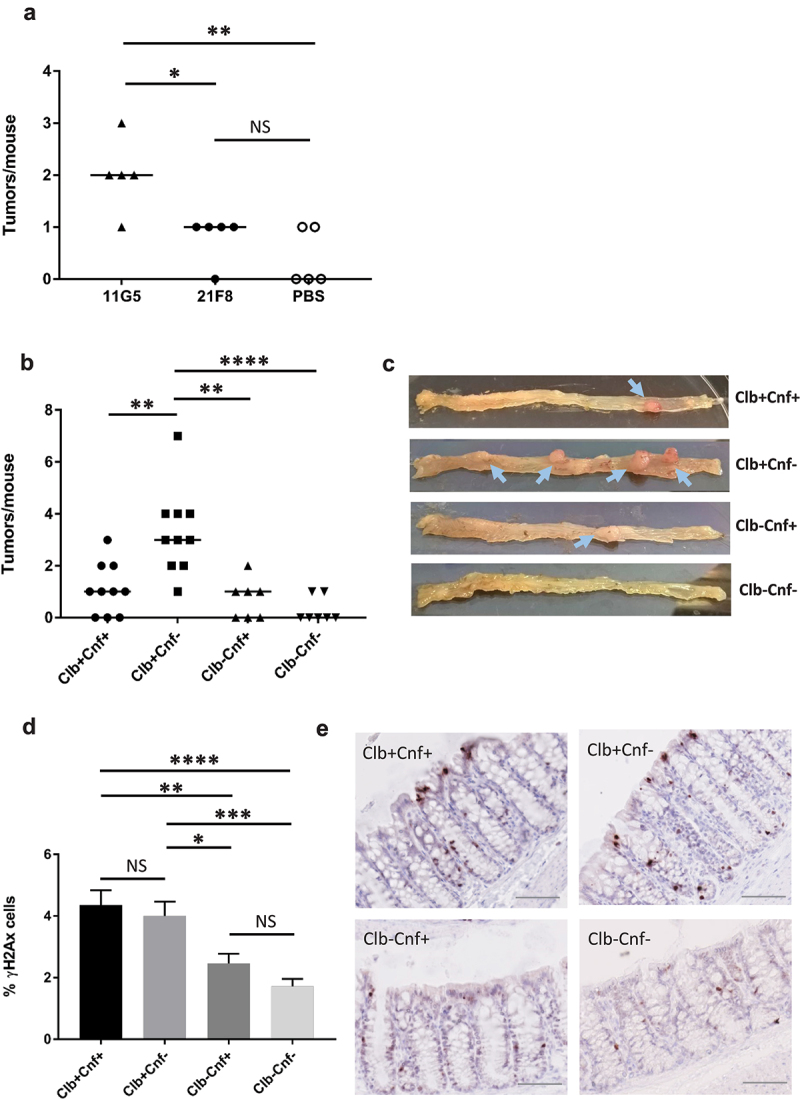
The absence of *cnf1* in CoPEC increases tumor development in *Apc^Min/+^* mice. *Apc^Min/+^* mice were treated with streptomycin for 3 days and then received water for 24 hours. (a) Mice orally received PBS (Day 0) or 10^9^ colony-forming units (CFU) of 11G5 or 10^9^ CFU of wild-type 21F8 (Clb+Cnf+) bacteria. (b–e) *Apc^Min/+^* mice were treated with streptomycin for 3 days and then received water for 24 hours. Mice were orally inoculated with wild-type 21F8 (Clb+Cnf+) or 21F8 mutants: Clb+Cnf-, Clb-Cnf+ or Clb-Cnf-. (a-e) Mice were killed at 50 days post-infection. (a, b) the number of colorectal tumors was determined using a dissecting microscope. The data points represent actual values for each individual mouse, and the bars indicate median values. Data were combined from two independent experiments. (c) Representative images of the colons of the inoculated mice. Arrows show macroscopic tumors. (d) Representative images of γH2AX immunohistochemical staining of nontumoral colonic mucosa (scale bars: 50 µm) and (e) quantification of γH2AX-positive cells determined from nontumoral colonic mucosa. Data are presented as means ± SEMs. Statistical analysis: Kruskal–Wallis ANOVA (*P <0 .05, **P < 0.01, ***P < 0.001, ****P <0 .0001).

C57BL/6 *Apc*^Min/+^ mice were gavaged with the 21F8 or 11G5 strain to assess the protumorigenic roles of the CoPEC strains. As expected, the number of colonic tumors increased in mice infected with the 11G5 strain compared to that in the uninfected mice (Figure 1a). Surprisingly, the number of tumors in the mice colonized with the 21F8 strain did not increase relative to that in the uninfected mice (Figure 1a). To evaluate the role of CNF1 in intestinal tumorigenesis, we generated isogenic mutants defective in CNF1 and/or colibactin (Clb) production, which were designated Clb+Cnf- for the mutant defective in CNF-1, Clb-Cnf+ for the mutant defective in colibactin, and Clb-Cnf- for the mutant defective in CNF1 and colibactin. The wild-type CoPEC 21F8 strain (Clb+Cnf+) and the three mutants were administered orally by gavage to *Apc*^*Min/+*^ mice (Figure 1b, c). Mice inoculated with Clb+Cnf- developed a significantly higher number of colonic tumors than mice inoculated with the isogenic mutants devoid of colibactin (Clb-Cnf+ and Clb-Cnf-). As an increase in the number of tumors may be due to an overabundance of Clb+Cnf- in the gut microbiota, the abundance of 21F8 wild-type or isogenic mutant strains in feces and colon biopsies was determined. We did not observe a significant increase in the bacterial load in the *Apc*^*Min/+*^ mice fed Clb+Cnf- (Supplemental Figure S2).

To test whether Clb+Cnf+ and Clb+Cnf- expressed colibactin and induced DNA damage *in vivo*, we assessed the occurrence of DSB in colonic epithelial cells by detecting the S139 phosphorylation on the histone H2AX (γH2AX), a well-known DSB marker^40^. Colonic biopsies revealed a significant increase in the number of γH2AX-positive cells in the mice exposed to the CoPEC strains Clb+Cnf+ and Clb+Cnf- compared to that in the mice exposed to the isogenic mutants Clb-Cnf+ and Clb-Cnf- (Figure 1d, e), showing that the *pks* island was functional and induced DNA damage of colonic epithelial cells. No significant differences in γH2AX staining in the colon of the mice inoculated with Clb+Cnf+ and Clb+Cnf- were observed (Figure 1d, e).

### The CoPEC 21F8 strain induces colibactin-dependent cytotoxicity in human colon cancer cells

**Figure 2.**
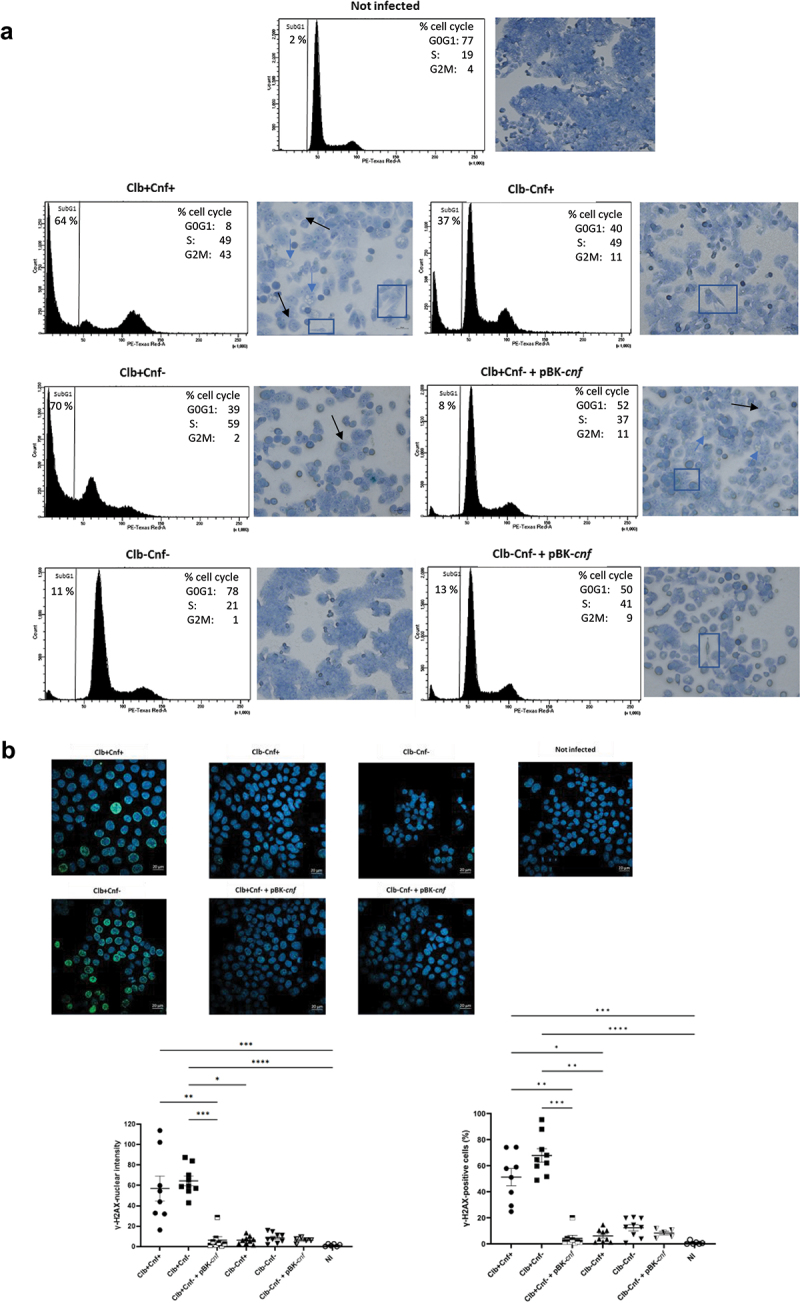
The *cnf1* gene modulates the genotoxic effect of CoPEC. HT-29 cells were infected for 3.5 h. (a) Cell cycle distribution and cytopathic effects were observed 72 h post-infection. Data are representative of two experiments. The dark and blue arrows show some megalocytes and multinucleated cells respectively as example, and rectangle surrounds the elongated cells. (b) Immunofluorescence of γH2AX indicating double strand breaks was assessed 24 h post-infection. The percentage of γH2AX-positive cells were counted from > 100 cell nuclei per well; the data points represent values for each individual cell wells. Alternatively, the average values of the measured nuclear fluorescence intensities was determined. Data are representative of two or three independent experiments and values are represented in mean ± SEM. Statistical analysis was performed by Kruskal–Wallis tests (NS, not significant, *P <0 .05, **P <0 .01, ***P < 0.001, ****P < 0.0001).

We next investigated the mechanisms by which CNF1 limits 21F8-induced colorectal tumorigenesis using cell cultures. To test whether *E. coli* 21F8 expresses the *pks* and *cnf1* genes and induces cytotoxicity, we infected human colon epithelial HT-29 cells with Clb+Cnf+ (21F8 strain), Clb-Cnf+, Clb+Cnf-, Clb-Cnf-, or with the corresponding trans-complemented mutants Clb+Cnf- +pBK*-cnf* and Clb-Cnf- +pBK*-cnf*. Colibactin and CNF1 are known to dysregulate cell cycle and induce cytopathic phenotypes. Transient infection with colibactin-producing bacteria (Clb+Cnf+ and Clb+Cnf-) caused cell cycle arrest, apoptosis induction and megalocytosis in HT-29 cells (Figure 2a, as expected.^19,41,42^ Clb-Cnf+ and Clb-Cnf- +pBK-*cnf* were able to induce accumulation of cells in both the S and G2/M phases compared to uninfected cells (Figures 2a and Supplemental Figure S3A). This was also found when cells infected with Clb+Cnf- were incubated with the purified toxin CNF1 (Supplemental Figure S3a). Infection with Clb-Cnf+, Clb-Cnf- +pBK*-cnf* and Clb+Cnf+ resulted in more flattened cells, elongated cells or cells spreading out compared with infection with Clb-Cnf- (Figure 2a), which are hallmarks of CNF1 cytopathic effects in epithelial cells.^34,41^ Only HT-29 cells infected with Clb+Cnf+ and Clb+Cnf- +pBK*-cnf* exhibited both megalocytosis and elongated cell morphologies. However, the cytopathic phenotype induced by Clb+Cnf- +pBK*-cnf* appears to be lower than those of Clb+Cnf+ (Figure 2a). Intriguingly, cells infected with this trans-complemented mutant exhibited a cell cycle similar to that of cells infected by Clb-Cnf+ or Clb-Cnf- +pBK*-cnf*. We quantified the transcription of three key *clb* genes involved in colibactin production in response to HT-29 cell infection. The mRNA levels of these genes were similar for Clb+Cnf+ and Clb+Cnf- (Supplemental Figure S4a), suggesting that the deletion of *cnf1* does not modify the production of colibactin.

Because the effects of colibactin results from DSB, γH2AX staining was monitored 24 hours post-infection (Figure 2b)^43^. Cells infected with strains not producing colibactin exhibited normal background levels of γH2AX (0.8 to 12%), whereas cells infected with Clb+Cnf+ or Clb+Cnf- showed strong γH2AX staining (51 *vs* 70%; Figure 2b). The γH2AX levels were not significantly different between those cells infected with Clb+Cnf+ or Clb+Cnf-. However, the incubation of purified CNF1 with HT-29 cells during Clb+Cnf- infection resulted in a ~ 50% reduction in γH2AX levels (Supplemental Figure S3d). Additionally, we unexpectedly found a drastic reduction in the number of γH2AX-positive cells infected by the trans-complemented mutant Clb+Cnf- +pBK*-cnf* in comparison with those infected with Clb+Cnf- (Figure 2b). The mRNA levels of *clbC*, *clbM* and *clbP* genes were significantly reduced (≥50%) for Clb+Cnf- +pBK-*cnf* compared to the parent strain (Supplemental Figure S4b). However, deletion of the *cnf* gene did not modify mRNA levels of the colibactin-synthesis gene (Supplemental Figure S4a). These results therefore suggest that the reduction of γH2AX levels observed with the trans-complemented mutant may be due to the action of CNF1 combined with a modified production of colibactin. To confirm this effect of CNF1 on γH2AX levels, we incubated HT-29 cells with bleomycin, a well-known chemotherapy drug that induces DNA damage, including DSB. We observed that the level of γH2AX induced by bleomycin was reduced when CNF1 was concurrently added. In conclusion, in our experimental conditions, the presence of the *cnf1* gene did not appear to affect the genotoxicity of the 21F8 strain. However, we show that the CNF1 toxin is able to reduce the levels of γH2AX induced by DSB.

### Deletion of the cnf1 gene increases CoPEC-induced cellular senescence and IL-8 production

**Figure 3.**
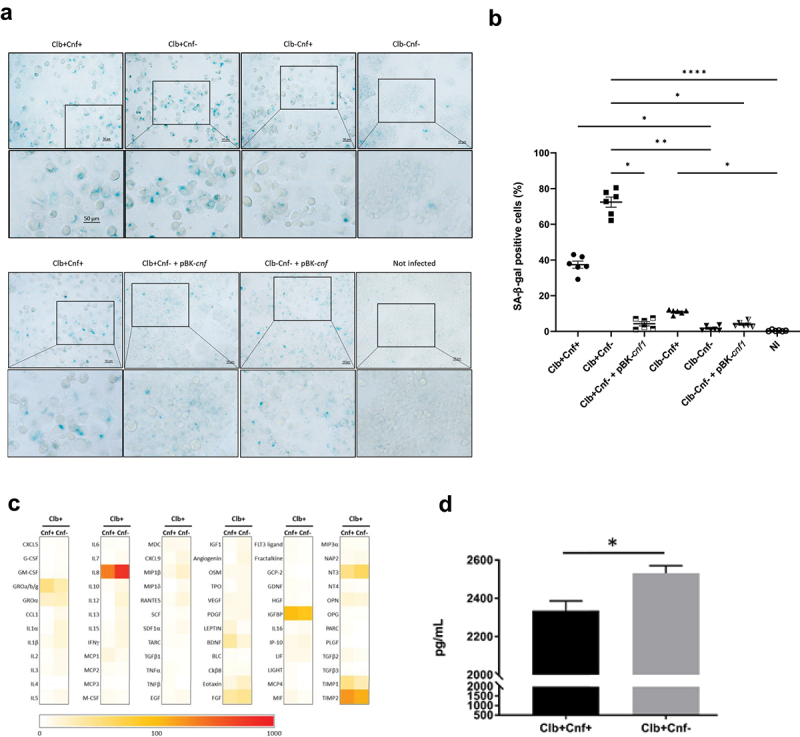
The *cnf1* gene decreases CoPEC-induced cellular senescence and IL-8 production. HT-29 cells were infected for 3.5 h. (a) on Day 3 post-infection, senescent cells were detected by β-galactosidase staining at pH 6. Representative images are shown (b) the percentage of senescence-associated β-galactosidase-positive cells was determined. The data points represent values the mean of fields with 100 to 200 cells for each individual cell wells (*n* = 3). Data are representative of two independent experiments and values are represented in mean ± SEM. (c) Heatmaps showing the relative rates of senescence-associated factors secreted by cells infected with Clb+Cnf+ or with the Clb+Cnf- mutant. Control values were set to 0 (negative control) and 100 (positive control). Orange-red indicates the predominant secreted factors. (d) IL-8 amounts secreted in culture supernatant by cells infected with Clb+Cnf+ or with the Clb+Cnf- mutant. The quantification of IL-8 was performed by ELISA. Data are representative of two independent experiments from three different conditioned media. Values represent means ± SEMs. Statistical analysis was performed by Kruskal–Wallis and Mann–Whitney tests (*P <0 .05, **P <0 .01, ****P < 0.0001).

CoPEC induces senescence of infected cells, leading to the secretion of inflammatory mediators and growth factors, which promote the proliferation of nearby uninfected cells.^22,44^ We infected HT-29 cells with Clb+Cnf+, Clb-Cnf+, Clb+Cnf-, Clb-Cnf-, or with the corresponding trans-complemented mutants Clb+Cnf- +pBK*-cnf* and Clb-Cnf- +pBK*-cnf* and detected senescent cells by staining for β-galactosidase at pH 6, a well-accepted senescence marker.^45^ Clb+Cnf+ infection increased the number of β-galactosidase-positive cells compared to that among uninfected cells or cells infected with colibactin-defective mutants (Clb-Cnf+, Clb-Cnf- and Clb-Cnf- +pBK*-cnf*) (Figure 3a,b). Although senescence-associated β-galactosidase (SA-β-gal) activity observed with Clb-Cnf+ (11%) was much lower than that of Clb+Cnf+ (37%), this activity was significantly increased compared to uninfected cells (0.4%), suggesting that CNF1 induces senescence as has been shown in other models.^46^ If we independently compare cells infected by Clb+Cnf- and Clb+Cnf+, the number of β-galactosidase-positive cells markedly increased when infected with Clb+Cnf- (72% *vs* 37%; *p = 0.0022;* Mann-Whitney test). The incubation of CNF1 with HT-29 cells during Clb+Cnf- infection also decreased the number of positive cells (Supplemental Figure S3c). P16 is a cell cycle gene that negatively regulates cell proliferation and is involved in pathways regulating senescence-mediated arrest. The number of p16-positive cells markedly increased when cells were infected with Clb+Cnf- compared to that observed in Clb+Cnf+ infected cells (Supplemental Figure S5). These results showed that the presence of CNF1 limited CoPEC-induced cellular senescence.

Next, we analyzed the senescence-associated secretory phenotype (SASP), which is known to underlie the pro-proliferative effect of colibactin.^22^ As expected, conditioned medium derived from cells infected with Clb+Cnf+ or Clb+Cnf- enhanced the proliferation of uninfected cells compared with conditioned medium derived from cells infected with Clb-Cnf+ or Clb-Cnf- (Supplemental Figure S6). However, we observed no significant difference in the pro-proliferative effect of conditioned medium derived from cells infected with Clb+Cnf+ or Clb+Cnf-, showing that *cnf1* did not modify the pro-proliferative effect mediated by CoPEC-induced cellular senescence *in vitro* (Supplemental Figure S6). Conditioned medium derived from HT-29-infected cells was then probed using an antibody array targeting 72 senescence-associated secreted factors (Figure 3c). In agreement with the results regarding the pro-proliferation effect, the production of growth factors was similar in the cells infected with Clb+Cnf+ and Clb+Cnf-. Interestingly, IL-8 production levels were the highest in cells infected with the Clb+Cnf- mutant. The difference in IL-8 production in the Clb+Cnf- infected cells was confirmed by ELISA (Figure 3d).

Overall, the presence of *cnf1* did not significantly modify the pro-proliferative effect of the colibactin-induced SASP in uninfected cells. However, it affected the induction of senescence mediated by colibactin and induced a subtle modification of the SASP, notably a decrease in the secretion of the proinflammatory cytokine IL-8.

### The E.coli 21F8 strain lacking cnf1 induces an increase in colonic inflammation and senescence in Apc^Min/+^ mice

**Figure 4.**
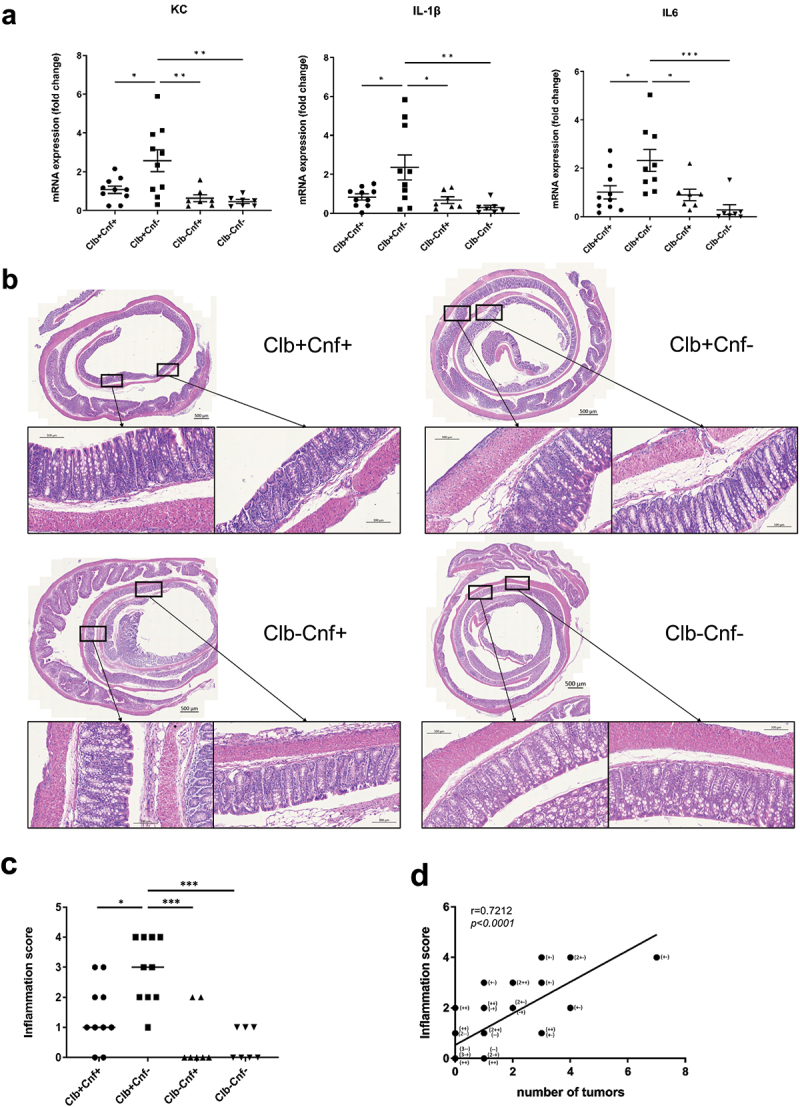
The *cnf1* gene limits CoPEC-induced colonic inflammation in *Apc^Min/+^* mice. *Apc^Min/+^* mice were orally administered 10^9^ colony-forming units (CFU) of wild-type *E. coli* 21F8 (Clb+cnf+) or 10^9^ (CFU) of its isogenic mutants: Clb+Cnf-, Clb-Cnf+ and Clb-Cnf-. Mice were killed on Day 50 after administration. (a) *Kc, Il-6, Tnf-α,* and *Il-1β* mRNA levels in the colonic mucosa were quantified by Qrt – PCR. The data points represent values for each individual mouse. Data are presented as means ± SEMs. (b) Representative images of H&E-stained colonic sections showing submucosal edema and inflammatory cell infiltration. (c) Inflammation score for each individual mouse, with the bars indicating median values. (d) Correlation between the inflammation score and the number of tumors [Clb+cnf+ (++, n = 10); Clb+Cnf- (+ -, n = 10); Clb-Cnf+ (- +, n = 7) and 7 Clb-Cnf- (- -, n = 10)]. The given *r* values indicate Spearman’s rank correlation, and the *P* value represents the significance of the test result. Statistical analysis was performed by the Kruskal–Wallis test (*P < 0.05, **P < 0.01, ***P <0 .001, ****P < 0.0001).

Given the differences in senescence and SASP observed in our *in vitro* assays, we investigated inflammatory responses in infected *Apc*^*Min/+*^ mice. We analyzed the expression of several pro-inflammatory factors by qRT – PCR in the colon of *Apc*^*Min/+*^ mice. Pro-inflammatory gene mRNA levels, including those of *KC*, the murine homolog of human IL-8, were significantly higher in mice inoculated with Clb+Cnf- than in those inoculated with Clb+Cnf+, Clb-Cnf+, or Clb-Cnf- (Figure 4a). These results were corroborated by histological analyses of colonic biopsies from *Apc*^*Min/+*^ mice. Colon sections from the Clb+Cnf- inoculated *Apc*^*Min/+*^ mice showed submucosal edema and cellular infiltration (neutrophils and mononuclear cells), whereas colon sections from the Clb+Cnf+-inoculated *Apc*^*Min/+*^ mice showed only few inflamed areas with weak inflammatory cellular infiltrate (Figures 4b and Supplemental Figure S7). Accordingly, the colonic inflammation score was significantly increased in *Apc*^*Min/+*^ mice inoculated with Clb+Cnf- compared to that in those inoculated with Clb+Cnf+ or mutants defective in colibactin production (Clb-Cnf+ and Clb-Cnf-) (Figure 4c). The degree of inflammation induced by Clb+Cnf- remained low, with no ulcers or extensive crypt damage. The increase in inflammation was in accordance with the increase in the tumor number observed in the mice inoculated with the Clb+Cnf- strain compared to that of the mice inoculated with the Clb+Cnf+ and Clb-Cnf+ mutants (Figure 1b). We observed a significant positive correlation between the inflammation score and the number of colonic tumors in the *Apc*^*Min/+*^ mice (Figure 4d), suggesting that CNF1 decreased colibactin-mediated colon tumorigenesis by inhibiting inflammation.

**Figure 5.**
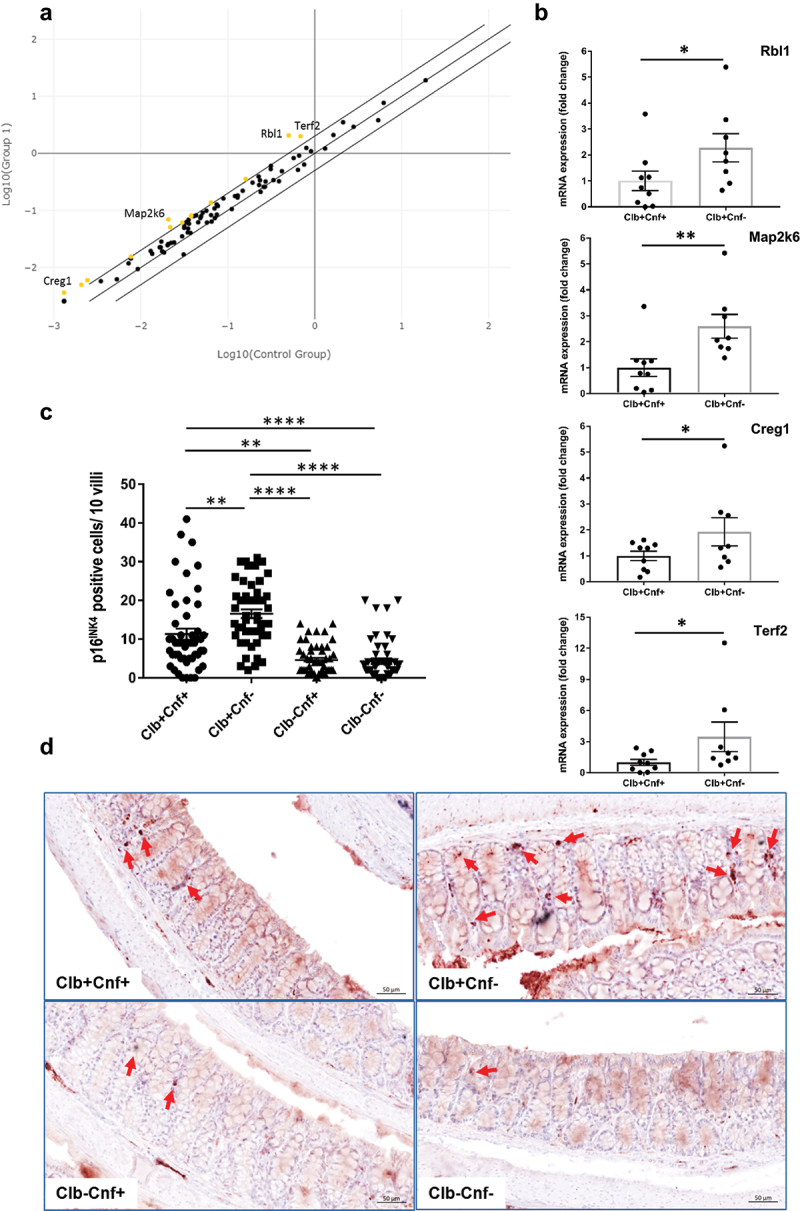
The *cnf1* gene limited CoPEC-induced senescence in the colon of *Apc^Min/+^* mice. *Apc^Min/+^* mice were orally administered 10^9^ colony-forming units (CFU) of wild-type *E. coli* 21F8 (Clb+Cnf+) or 10^9^ (CFU) of its isogenic mutants: Clb+Cnf-, Clb-Cnf+ and Clb-Cnf-. Mice were killed on Day 50 after administration. (a) Scatter plot of differential gene expression in the colonic mucosa of mice inoculated with Clb+Cnf- and Clb+Cnf+ determined using a cellular senescence RT2 Profiler PCR Array (the conditioned medium used was a mix of 3 replicates of mice infected by Clb+Cnf+ or Clb+Cnf- strains). The yellow circles show ≥ 2-fold upregulated genes in the mice inoculated with Clb+Cnf- compared to those in mice inoculated with Clb+Cnf+. (b) Relative *Rbl1, Map2k6, Creg1* and *Terf2* mRNA levels quantified by Qrt – PCR (RT2 Profiler PCR) in the colonic mucosa of mice inoculated with Clb+Cnf- or Clb+Cnf+. The data points represent values for each individual mouse (c) Quantification of p16^INK4a^-positive cell number/10 villi determined from 100 villi/mouse and 5 mice/group. Data are presented as means ± SEMs. Statistical analysis was performed using the Kruskal–Wallis test (*P < 0.05, **P <0 .01, ****P <0 .0001). (d) Representative images of immunohistochemical expression of p16^IN4a^ in *Apc^Min/+^* colonic mucosa of infected mice. The arrowheads show positive cells (scale bars: 20 µm).

Next, we investigated whether the presence of the *cnf1* gene limited CoPEC-induced senescence *in vivo*. A PCR array designed to analyze a panel of 84 genes associated with senescence was performed with RNA extracted from colonic biopsy samples of mice colonized by Clb+Cnf+ or by its mutant Clb+Cnf-. Twelve genes were ≥ 2-fold upregulated in the Clb+Cnf- inoculated group compared to the group inoculated with Clb+Cnf+ (Figure 5a). qRT – PCR tests confirmed the upregulation of the most deregulated genes (Figure 5b), including *Creg-1*, an enhancer of the p16^INK4a^-dependent senescence pathway.^47^ Accordingly, there was a significant decrease in p16^INK4a^-positive cells in the murine colonic tissues colonized with Clb+Cnf+ compared to those in the murine colonic tissues colonized with Clb+Cnf- (Figure 5c), with only a few p16^INK4a^-positive cells detected in the colonic epithelium of the mice inoculated with the mutants defective in colibactin production (Figure 5d). Overall, these results suggested that CoPEC induces senescence in the colon of *Apc*^*Min/+*^ mice and that CNF1 limits this process.

We investigated the fecal microbiota composition by 16S rRNA gene sequencing. In addition to increases CoPEC-induced cellular senescence and inflammation, the deletion of *cnf1* gene was associated with a change in intestinal microbiota composition (these results are detailed in the supplementary data).

### The CNF1 toxin impairs the development of colon tumors in Apc^Min/+^ mice

**Figure 6.**
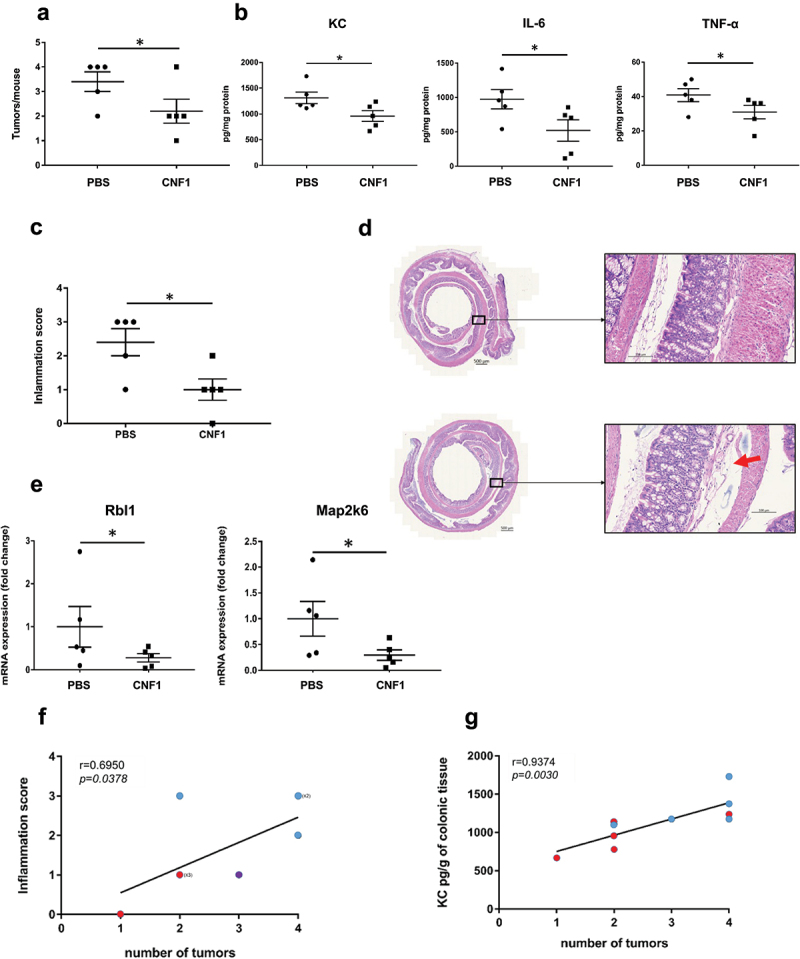
In CoPEC-infected *Apc^Min/+^* mice, intrarectal administration of CNF1 impedes the development of colon tumors and decreases inflammation. *Apc^Min/+^* mice were orally administered 10^9^ colony-forming units of the *E. coli* 21F8 mutant producing colibactin but defective in *cnf1.* Two days post-infection, the mice received an intrarectal injection of 10 µg of CNF1 protein or PBS every 7 days for 7 weeks. (a) the number of colorectal tumors by mouse was determined using a dissecting microscope. The data points represent actual values for each individual mouse, and the bars indicate median values. (b) the levels of secreted cytokines (KC, IL-6 and TNF-α) in colonic tissue were quantified by ELISA. (c) Inflammation score is presented as the mean ± SEM. (d) Representative images of H&E-stained colonic mouse sections of PBS- or CNF1-treated mice. The red arrow shows submucosal edema. e) *Rbl1* and *Map2k6* mRNA relative levels in colonic mucosa were quantified by Qrt – PCR (RT2 Profiler PCR). (f) Correlation between the inflammation score and the tumor number. (g) Correlation between the KC levels in colonic tissue and the tumor number. Blue dots represent mice treated with PBS (n = 5) and red dots represent those treated with CNF1 (n = 5). Statistical comparisons were carried out by unpaired t test (*P <0 .05) after normality testing. Spearman correlation analysis was performed between the inflammation score or KC concentration and the tumor number. The given *r* values indicate Spearman’s rank correlation, and the *P* value represents the significance of the test result.

To confirm the importance of CNF1 in preventing the tumorigenic activity of colibactin, we investigated the impact of weekly rectal administration of the CNF1 protein on the development of colonic tumors in *Apc*^*Min/+*^ mice colonized by pro-tumorigenic Clb+Cnf-. We observed no difference in intestinal colonization by Clb+Cnf- between the mice treated with CNF1 and the mice treated with a saline solution (PBS) (Supplemental Figure S8). The mice treated with CNF1 had significantly fewer tumors than the PBS-treated mice (Figure 6a). The decrease in tumor number was associated with a reduction in both inflammation and senescence marker levels (Figure 6b–e). Inflammatory cell infiltration and submucosal edema were significantly less pronounced in mice treated with CNF1 than in PBS-treated mice (Figure 6d). Accordingly, there was a significant decrease in the histological colonic inflammation score of the *Apc*^*Min/+*^ mice colonized with *E. coli* Clb+Cnf- and treated with CNF1 in comparison to that of PBS-treated mice (Figure 6c). As shown in (Figure 6f, g) the histological inflammation score and KC rate were significantly and positively correlated with the number of tumors in the *Apc*^*Min/+*^ mice. The mice with the highest number of tumors and the highest level of colonic inflammation were those that did not receive CNF1. This observation was found with *Apc*^*Min/+*^ mice colonized by pro-tumorigenic 11G5 (Supplemental Figure S9). Overall, these results demonstrated that CNF1 limited the development of colonic tumors in CoPEC-infected *Apc*^*Min/+*^ mice, like by decreasing senescence and/or chronic low-grade inflammation induced by CoPEC.

## Discussion

Colibactin and CNF1, two common toxins of *E. coli* that affect the eukaryotic cell cycle, are putative pro-tumorigenic factors.^20,22,29,36,37,48^ Our epidemiological data demonstrated that CNF1 is almost always associated with colibactin in *E. coli* strains isolated from CRC patients. We thus hypothesized that coproduction of these two cyclomodulins might enhance colorectal carcinogenesis. However, our results show that a human CoPEC strain coproducing colibactin and CNF1 did not exhibit pro-tumorigenic activity in *Apc*^*Min/+*^ mice, unlike human *E. coli* strain 11G5, which produces only colibactin. We constructed isogenic mutants defective for CNF1 and/or colibactin production from the clinical strain 21F8. We observed independent impacts of CNF1 and colibactin on eukaryotic cells in terms of cytopathic effects that were in agreement with their production from independent genetic structures.^49^ Interestingly, isogenic deletion of *cnf1* in 21F8 revealed the pro-tumorigenic activity of the strain, and rectal administration of CNF1 in *Apc*^*Min/+*^ mice colonized by the 21F8 mutant defective in *cnf1* decreased colonic tumor development. Counterintuitively, these results revealed that CNF1 hinders tumorigenesis induced by colibactin-producing *E. coli* in the *Apc*^*Min/+*^ CRC model. Further analyses highlighted the underlying mechanisms.

The carcinogenic effect of CNF1 has previously been linked to its ability to promote Rho GTPase-dependent cellular effects, proinflammatory NF-kB pathway activation, cell growth and apoptosis suppression, tumor invasiveness, epithelial-to-mesenchymal transition (EMT) and metastasis.^30,32,36,37,48,50,51^ However, *in vitro* and *in vivo* studies have demonstrated the anti-proliferative and cytotoxic effects of CNF1 in cancer cell lines,^52–55^ suggesting a two-sided paradigm of CNF-1 impact.^48^ In the present study, isogenic *E. coli* 21F8 producing only CNF1 did not induce metastasis or enhance colonic tumorigenesis in the *Apc*^*Min/+*^ murine model of colon cancer. CNF1 behaved therefore less as an anti-tumorigenic factor than as a protective factor against colibactin.

We observed both *in vitro* and *in vivo* that CNF1 reduced colibactin-induced (i) inflammatory cell infiltration, (ii) senescence, and (iii) senescence-associated secretion of the key pro-inflammatory cytokine, KC/IL-8. Senescence and the resulting SASP have been identified as cellular processes sustaining tumor development in a xenograft model.^22,56^ Chronic inflammation is known to be an important risk factor for numerous forms of cancer, including CRC,^57^,and inflammation has also been identified as a key player in colibactin tumorigenic activity *in vivo*.^16,22,58^ In addition, several studies have implicated IL-8 in the progression of various types of cancer,^59–63^ including CRC.^64,65^ CNF1 has pro-inflammatory activity in acute infections, such as urinary tract infections.^66^ In contrast, it may also counteract the overexpression of proinflammatory cytokines such as IL-8 during chronic colonization, as reported by Loizzo *et al*. in the context of the chronic inflammation associated with Alzheimer’s disease.^67^ Therefore, the impact of CNF1 on colibactin-induced tumorigenesis can be explained in the context of the tissue organization field theory. According to this theory, alterations in tissue organization by cellular processes such as inflammation or senescence lead to carcinogenesis.^68^ Additionally, the bacterium-engulfing activity of CNF1, linked to the activation of Rho GTPases by deamidation,^50,69^ could contribute to the observed phenotype. Upon expressing CNF1, the bacteria may acquire invasive capacities and thereby shelter from the host immune system, generating less inflammation.^29,69^ Thus, CNF1 appears to function as a protective factor against colibactin, impeding the emergence of a microenvironment and inflammatory cell infiltration promoting tumorigenesis in the *Apc*^*Min/+*^ mouse model.

On the other hand, double-strand DNA breaks are the primary effect of colibactin and induce oncogenic mutations in human CRC.^18,20^ Our results showed that the presence of *cnf1* gene under normal conditions, *i.e* when not overexpressed, does not modify the intensity of colibactin-induced DNA damage. However, the capacity to repair injury may be different in presence of CNF1. In this regard, the level of γH2AX was considerably reduced when the cells were infected with the Clb+Cnf- strain trans-complemented with *cnf1*. Rho GTPases such as Rho and Rac proteins, that are involved the regulation of DNA repair systems,^70^ are the target of CNF1, which induces their constitutive overactivation through the deamination of a specific glutamine residue in the infected cells. A high Rho GTPases activity have been directly correlated with a high level of DNA repair, and inhibition of Rho GTPases dramatically reduces γH2AX and the formation of DNA damage foci.^70^ Furthermore, CNF1-induced Rac1 activation positively regulates RhoB expression cultures of epithelial cell lines, including HT-29 cells.^71^ CNF1 may therefore enhance DNA repair and then weaken the carcinogenic effect of colibactin.

In addition, there is emerging evidence for a cancer suppressive role for RhoB through inhibitory effects on cell proliferation, survival, invasion and metastasis.^72^ These *in vitro* observations were supported by *in vivo* findings. RhoB-depleted cells form tumors more efficiently than cells expressing RhoB when injected intraperitoneally into mice.^73^ Cells transfected with RhoB and subcutaneously implanted into nude mice suppress tumor growth.^74^ CNF1-induced activation of Rho GTPases may thereby hinder colon tumorigenesis induced by colibactin-producing *E. coli*. However, Rho GTPases interact with a wide range of effectors and cellular signaling cascades. Their role in cancer is dependent of cellular context and they can also contribute to tumor formation.^72^ Further studies are required to decipher the features that determine the impact of Rho GTPases in carcinogenesis.

The findings of this study underline the strong epidemiological link between CNF1 and colibactin in *E. coli* strains associated with human CRC. In this study, more than 90% of *E. coli* strains harboring the *cnf1* gene carried the genomic island *pks* producing colibactin. This finding is in accordance with previous reports that demonstrated this association in uropathogenic *E. coli* (83%) and *E. coli* isolated from fecal samples of different animals (62–100%).^41,75,76^ Thus, the association of CNF1 and colibactin is common in *E. coli*, which is the predominant aerobic organism observed in the gastrointestinal tract and common in the human intestinal microbiota (i.e., >90%).^77^ The prevalence of patients colonized with strains of *E. coli* bearing both the *pks* genomic island and *cnf1* gene (Table 1) was not significantly higher in CRC patients than in controls (24%, *n* = 19/80 *versus* 14%, *n* = 4/28; *p = 0.432*). Thus, the frequent association of CNF1 with colibactin in *E. coli*, an extremely common bacterium in the gut, does not result in higher incidence of CRC.

In conclusion, we found that the *pks* island and *cnf1* gene are frequently co-harbored in *E. coli*. Our work showed for the first time that CNF1 hinders CoPEC-induced colorectal carcinogenesis by decreasing CoPEC-induced cellular senescence and inflammation. The presence of *E. coli* strains producing only colibactin might represent a higher risk of CRC than the presence of strains producing both CNF1 and colibactin. Another finding that emerges from this study is the need to consider the genetic diversity of bacteria colonizing CRC patients and especially their virulome to understand microbiota-induced carcinogenesis and to determine whether the bacterium is deleterious to the host.

## Materials and methods

Information on the bacterial strains, cell culture, infection assays, CoPEC colonization, quantitative reverse transcription polymerase chain reaction (qRT – PCR), antibody array chips, enzyme-linked immunosorbent assays (ELISAs), senescence-associated β-galactosidase staining, immunofluorescence microscopy, histological observations, and immunohistochemical staining appears in the Supplementary materials.

### Bacterial strains and construction of isogenic mutants

The clinical *E. coli* 11G5 and 21F8 strains were isolated from tumors of patients with CRC. The following isogenic mutants of the 21F8 strain were generated using the method described by Datsenko *et al* . ^78^ and modified by Chaveroche *et al*. ^79^ 21F8Δ*cnf* (Clb+Cnf-) with deletion of the *cnf1* gene, 21F8Δ*clbQ* (Clb-Cnf+) with deletion of the *clbQ* gene of the *pks* island and 21F8Δ*cnf*Δ*clbQ* (Clb-Cnf-) with deletion of the *cnf1* and *clbQ* genes. The ClbQ thioesterase regulates colibactin synthesis and consequently its genotoxic activity. CoPEC strains deficient in *clbQ* are unable to produce functional colibactin.^22^ In brief, the method consisted of the replacement of the gene of interest by a selective antibiotic cassette (kanamycin) generated by PCR using primers reported in the Table S1 from supplementary materials. The hemolysin A (*hlyA*) was deleted from the 21F8 strain and its isogenic mutants with the same method to avoid lysis of the HT-29 cells. The presence of deletions and the absence of additional genetic modifications were verified by sequencing the clinical 21F8 strain and its isogenic mutants. With the In-Fusion^Ⓡ^ HD Cloning (Takara), *cnf1* gene was cloned into the pBK-CMV plasmid (Table S1). The 21F8Δ*hlyA*Δ*cnf and* 21F8Δ*hlyA*Δ*cnf*Δ*clbQ* was electroporated with sequenced pBK-CMV-*cnf1* plasmid. For experiments, strains were growth in Luria-Bertani (LB) broth overnight at 37°C with 110 rpm agitation. All the strains for this study were summarized in Table S2.

### *Colonization of the* Apc^Min/+^ *murine model*

C57BL/6 *Apc*^Min/+^ females (6–7 weeks of age) were used. The mice were inoculated as previously described.^23^ All the mice were sacrificed 50 days post-infection. Colonic tumor number and tumor volume ([width^2^ × length]/2) were determined using a dissecting microscope. Colonic tissue adjacent to tumors were fixed in buffered 4% formalin and embedded in paraffin. Non-tumoral colonic mucosa was frozen at −80°C for protein and RNA extraction. For the experiment employing rectal administration of the CNF1 protein, *Apc*^*Min/+*^ mice were inoculated as previously described^23^ and then received an intrarectal injection of 10 µg of CNF1 protein or PBS under anesthesia with isoflurane. The injections were administered 2 days post-infection and then once per week for 7 weeks. CNF1 was purified as described previously.^51^

### Ethical statement

Animal protocols were in accordance with French and European Economic Community guidelines (86–60, EEC) for the care of laboratory animals. The study was approved by the French Ministry of Higher Education Research and Innovation (Apafis no. 22798).

Biological samples were collected from CRC patients (ethical approval for human study no. DC-2017–2972). All patients underwent surgery for resectable CRC in the Digestive and Hepatobiliary Surgery Department of the University Hospital of Clermont-Ferrand.^38^ All patients were adult volunteers and signed an informed consent form before inclusion in the study. The exclusion criteria included clinically suspected hereditary CRC based on the revised Bethesda criteria, neoadjuvant chemotherapy receipt, a history of previous colonic resection, emergency surgery, and use of antibiotics within 4 weeks before the surgery.

### RT^2^ Profiler PCR Array

Eighty-four genes or biological pathways involved in cellular senescence were analyzed using the RT^2^ Profiler PCR Array Mouse Cellular Senescence system (PAMM-050Z; Qiagen, Maryland, USA). According to the manufacturer’s protocol, real-time PCR was performed using RT^2^ Profiler PCR Arrays in combination with RT^2^ SYBR Green PCR Master Mix (Qiagen, Maryland, USA) using a mixture of cDNA obtained from three colonic biopsy samples of mice colonized by 21F8 (Clb+Cnf+) or its mutant Clb+Cnf-. The expression levels of the 84 genes were quantified relative to the values obtained for housekeeping genes (*ACTB*, *B2M* and *GAPDH*). Data analyses were performed using web-based analysis software (http://pcrdataanalysis.sabiosciences.com/pcr/arrayanalysis.php). In addition, we further performed a similar RT^2^ Profiler PCR assay using custom plates including four genes (*RBL1*, *MAP2K6*, *CREG1* and *TERF2*) in addition to the housekeeping genes (*ACTB*, *B2M* and *GAPDH*). The analysis included five or six mice in each group (mice with intrarectal injection of CNF1 protein or PBS or mice colonized by Clb+Cnf+, Clb+Cnf-, Clb-Cnf+ or Clb-Cnf-).

### Statistical analysis

GraphPad Prism software was used for all statistical calculations. Data comparisons with multiple groups were analyzed by one-way Kruskal – Wallis test. Data comparisons between 2 groups were performed with unpaired t test or a Mann-Whitney U-test depending on the normality test. Spearman’s correlation analysis was performed for correlation testing. A value of *P* <0 .05 was considered to indicate a statistically significant difference.

## Supplementary Material

Supplemental Material

## Data Availability

The data that support the findings of this study are available in Mendeley data at http://doi.org/10.17632/zwck97zw4k.1.
