# Supplementary material for: Cytotoxic necrotizing factor 1 hinders colon tumorigenesis induced by colibactin-producing Escherichia coli in ApcMin/+ mice
Source: Gut Microbes. 2023 Jul 7;15(1):2229569. doi: 10.1080/19490976.2023.2229569 (PMC10332217; doi:10.1080/19490976.2023.2229569)
Supplement: Supplemental Material [file KGMI_A_2229569_SM4854.docx]

**Supplementary data**

**Cytotoxic necrotizing factor 1 hinders colon tumorigenesis induced by colibactin-producing *Escherichia coli* in *Apc^Min^*^/+^ mice.**

Héloïse Chat^1^, Guillaume Dalmasso^1^, Catherine Godfraind^1,2^, Virginie Bonnin^1^, Racha Beyrouthy^1^, Mathilde Bonnet^1,3^, Nicolas Barnich^1,3^, Amel Mettouchi^4^, Emmanuel Lemichez^4^, Richard Bonnet^1,5^, Julien Delmas^1,5*^

^1^ University Clermont Auvergne, Inserm U1071, INRAE USC 1382, Microbes, Intestin, Inflammation et Susceptibilité de l'Hôte (M2iSH), Centre de Recherche en Nutrition Humaine Auvergne, Clermont-Ferrand, 63001, France

^2^ Neuropathology Unit, University Hospital of Clermont-Ferrand, France

^3^ University Clermont Auvergne, Institut Universitaire de Technologie, Clermont-Ferrand, France

^4^ Institut Pasteur, University of Paris, CNRS UMR2001, Bacterial Toxins Unit, 75724 Paris, France

^5^ Department of Bacteriology, University Hospital of Clermont-Ferrand, France

* **Corresponding authors:** Julien Delmas, E-mail: [julien.delmas@uca.fr](mailto:julien.delmas@uca.fr), Phone: (33) 4 73754929. Mailing address: M2iSH, UMR 1071 Inserm, University of Clermont Auvergne, 28 Place Henri-Dunant, Clermont-Ferrand 63001, France.

**Supplementary figures**


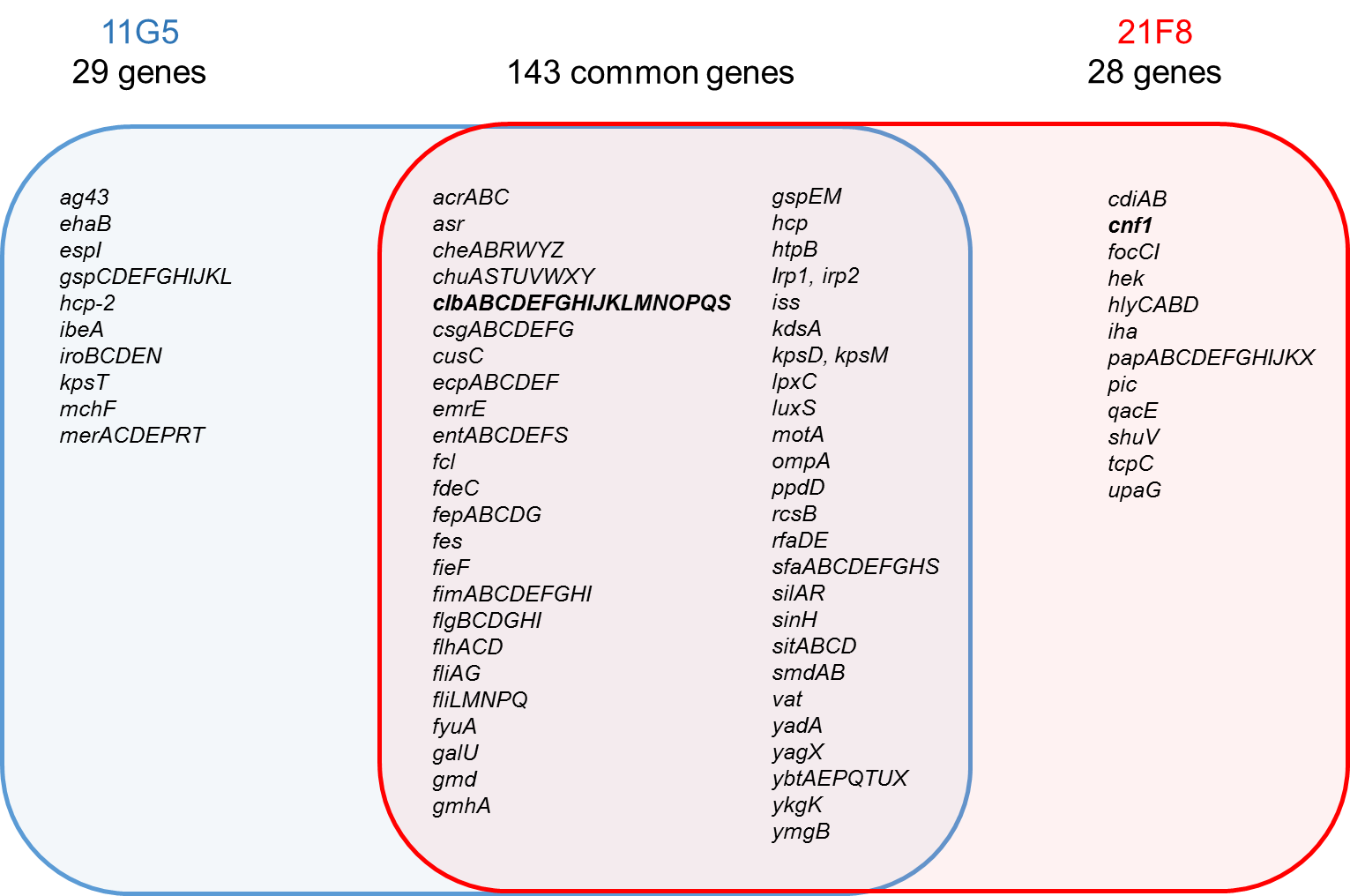


**Figure S1: Virulomes of the 11G5 and 21F8 strains extracted from whole genome sequences.**

The virulome was analysed with VirulenceFinder 2.0^1^. Diagrams indicate the overlapping genes between the 11G5 and 21F8 strains. The red line indicates the virulence genes of 21F8, and the blue line indicates the virulence genes of 11G5.

**
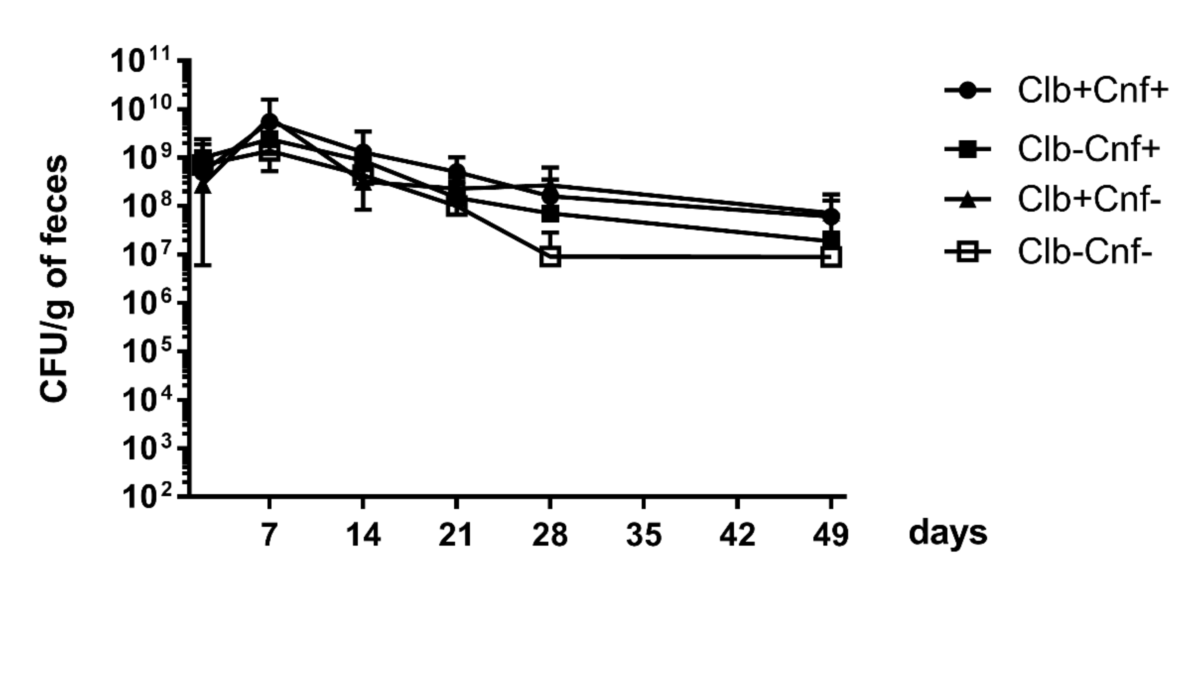
**

**A**

**
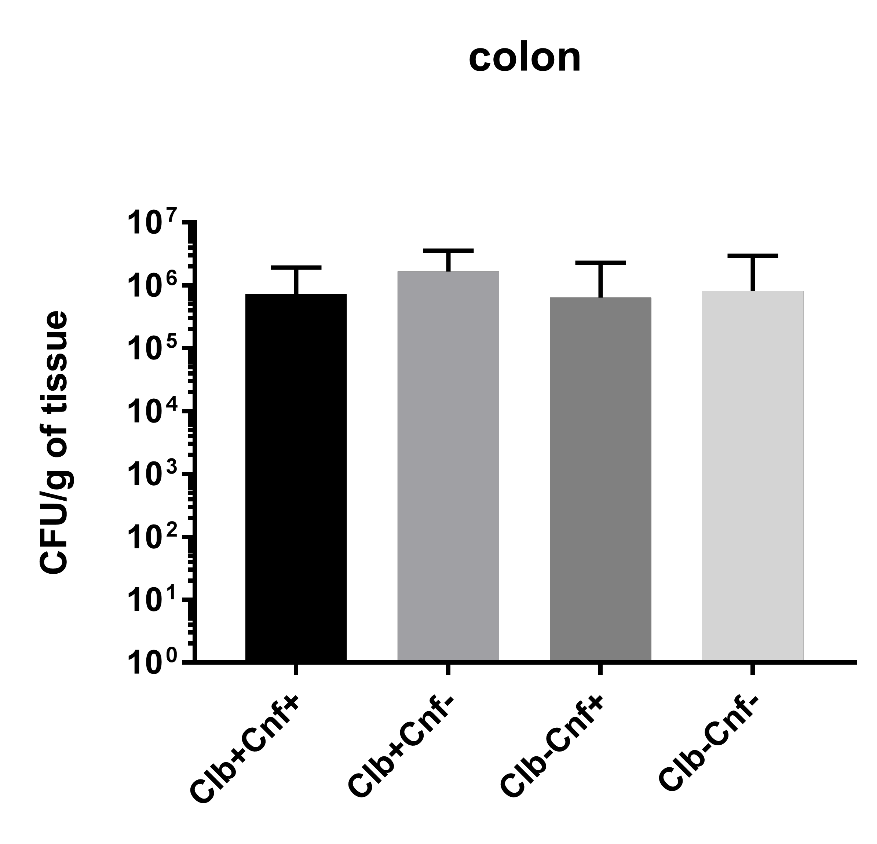
**

**B**

**Figure S2: No difference in colonization of *Apc^Min/+^* mice between the wild-type and mutant strains.** *Apc^Min/+^* mice were treated with streptomycin for 3 days and then received water for 24 hours. Mice were orally gavaged with wild-type 21F8 (Clb+Cnf+) or 21F8 isogenic mutants: 21F8*ΔCnf1* (Clb+Cnf-), 21F8*ΔclbQ* (Clb-Cnf+) or 21F8*ΔclbQΔCnf1* bacteria (Clb-Cnf-). Mice were killed 50 days post-infection. *(A)* Bacterial colonization in the stools of mice from 2 to 49 days post-infection. *(B)* *E. coli* 21F8 or 21F8 isogenic mutant colonization associated with nontumoral colonic tissue assessed at 50 days post-infection. Colonization data are presented as the means ± standard deviations. *(A-B)* are representative of 2 independent experiments.

**
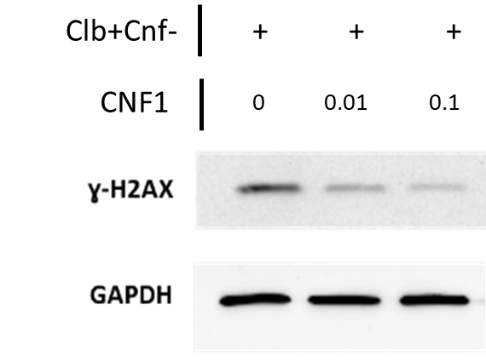
**

**D**

**Clb+Cnf- + CNF1**


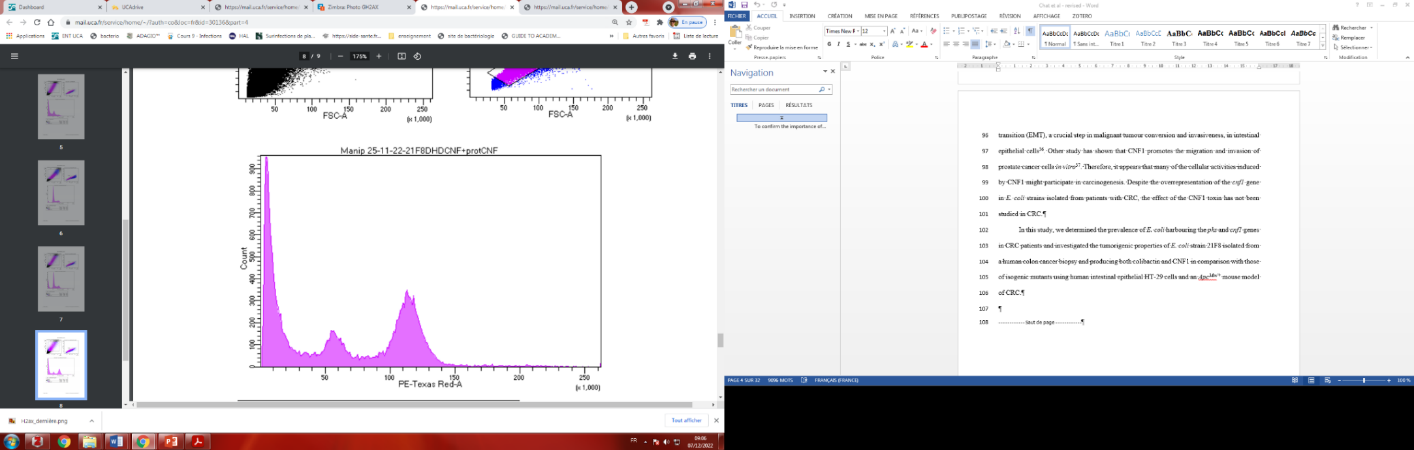


SubG1

54 %

% cell cycle

G0G1: 35

S: 33

G2M: 32

**Clb+Cnf-**


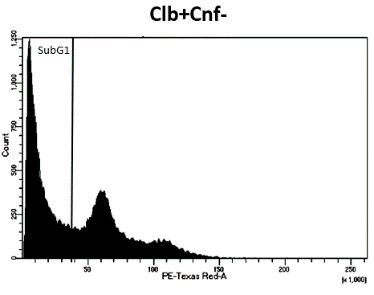


% cell cycle

G0G1: 39

S: 59

G2M: 2

**A**

70 %

**B**

**C**

**
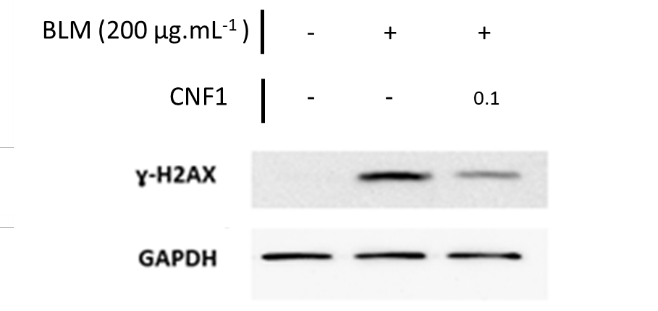
**

**E**


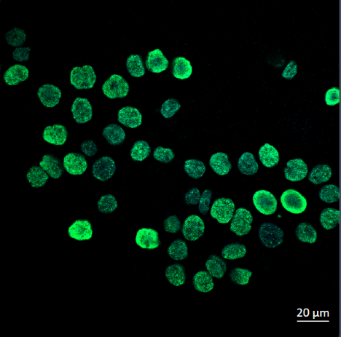


**Clb+Cnf-**


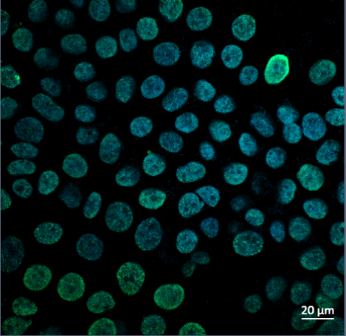

**F**

**19 ± 6%**


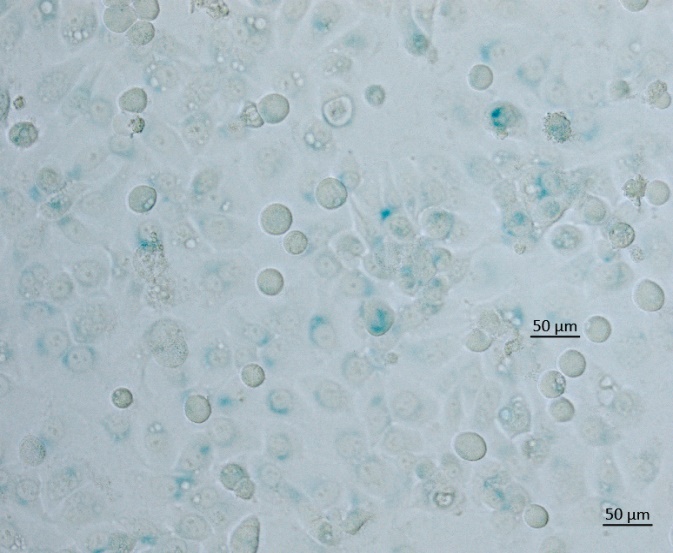


**Clb+Cnf- + CNF1**


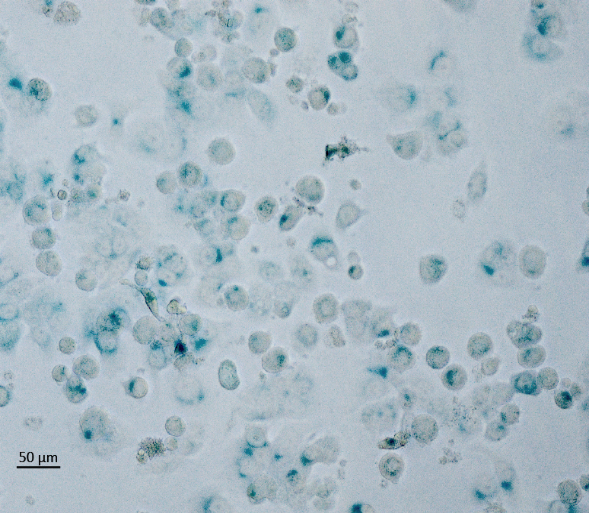


**74 ± 8%**

**Clb+Cnf-**

**Figure S3: CNF1 can modulate the toxic effect of CoPEC.** HT-29 cells were infected with Clb+Cnf- and incubated with the CNF1 toxin for 3.5 h. (A) Cell cycle distribution were observed 72 h after infection of HT-29 cells incubated with 0.1 nM CNF. (B) γH2AX-positive cells were assessed 24 h after infection of HT-29 cells incubated with different concentration of CNF1 (0.01 to 10 nM); means and standard error of the mean of results from at least two independent experiments are shown. (C) Representative images of γH2AX immunostaining in HT-29 cells infected for 3.5 h incubated or not with CNF1 (0.1 nM). (D-E) Immunoblot analysis of cell lysates extracted 24 h post infection. GAPDH was used as a loading control for cell lysates. Signal intensities of bands were measured using ImageLab software. γH2AX signals were corrected to account for any variation in loading using GAPDH signal intensity. (D) Experimental signal normalized to the “Clb+Cnf-” condition was set to 1 for each experiment. Data represents means ± SEMs corresponding to two experiments with two replicates. (E) HT-29 cells were incubated with bleomycin (BLM) at 200 µg.mL^-1^ with or without CNF1 (0.1 nM). Experimental signal normalized to the “BLM-treated” condition was set to 1 for each of the two experiments performed. (F) Cellular senescent were detected by β-galactosidase staining at pH 6 three days after infection of HT-29 cells incubated or not with CNF1 (0.1 nM).


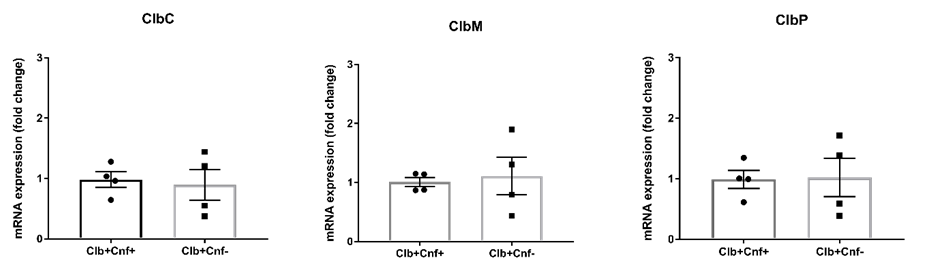


**B**

**A**

**Figure S4: Deletion of *cnf1* gene does not significantly impacted transcription of colibactin genes in the 21F8 strain.**

mRNA expression of *clbC, clbM* and *clbP* genes involved in colibactin production was analysed from bacteria having infected the HT-29 cells. RT-qPCR were performed for 21F8Δ*hlyA* (Clb+Cnf+) and 21F8Δ*hlyA*Δ*cnf* (Clb+Cnf-) (A) or 21F8Δ*hlyA*Δ*cnf* (Clb+Cnf-) and 21F8Δ*hlyA*Δ*cnf* + pBK-*cnf1* (B). The data points represent values for each replicate. Data are presented as means ± SEMs. Statistical comparisons were carried out by Mann-Whitney U-test *(*P < .05*).


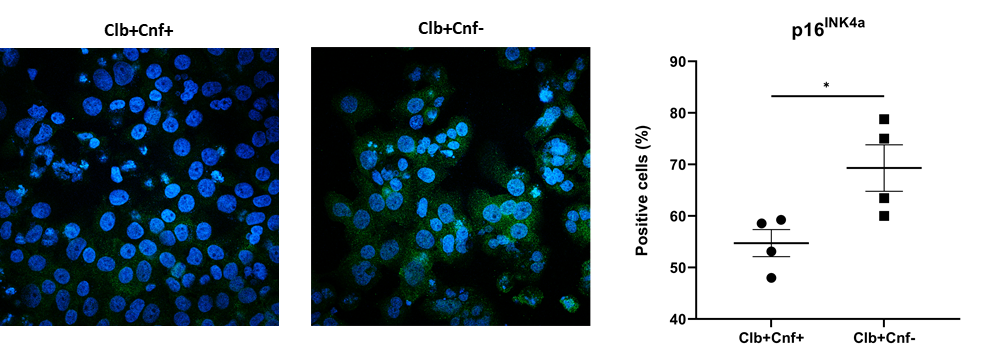


**Figure S5: CNF1 decreases the CDKN2A/p16I^NK4a^-senescence marker following CoPEC infection.** HT-29 cells were infected with the indicated *E. coli* 21F8 (Clb+Cnf+) or isogenic mutants (Clb+Cnf-) for 3.5 h. At day 3 post-infection, anti p16^INK4a^ immunofluorescence was performed to evaluate the percentage of p16^INK4a^-expressing cells were counted with QuPath software. Data are representative of two independent experiments with two replicates. Values represent means ± SEMs. The significance of differences was assessed by Kruskal–Wallis tests (NS, not significant; **P* < .05; *****P* ≤ .0001).


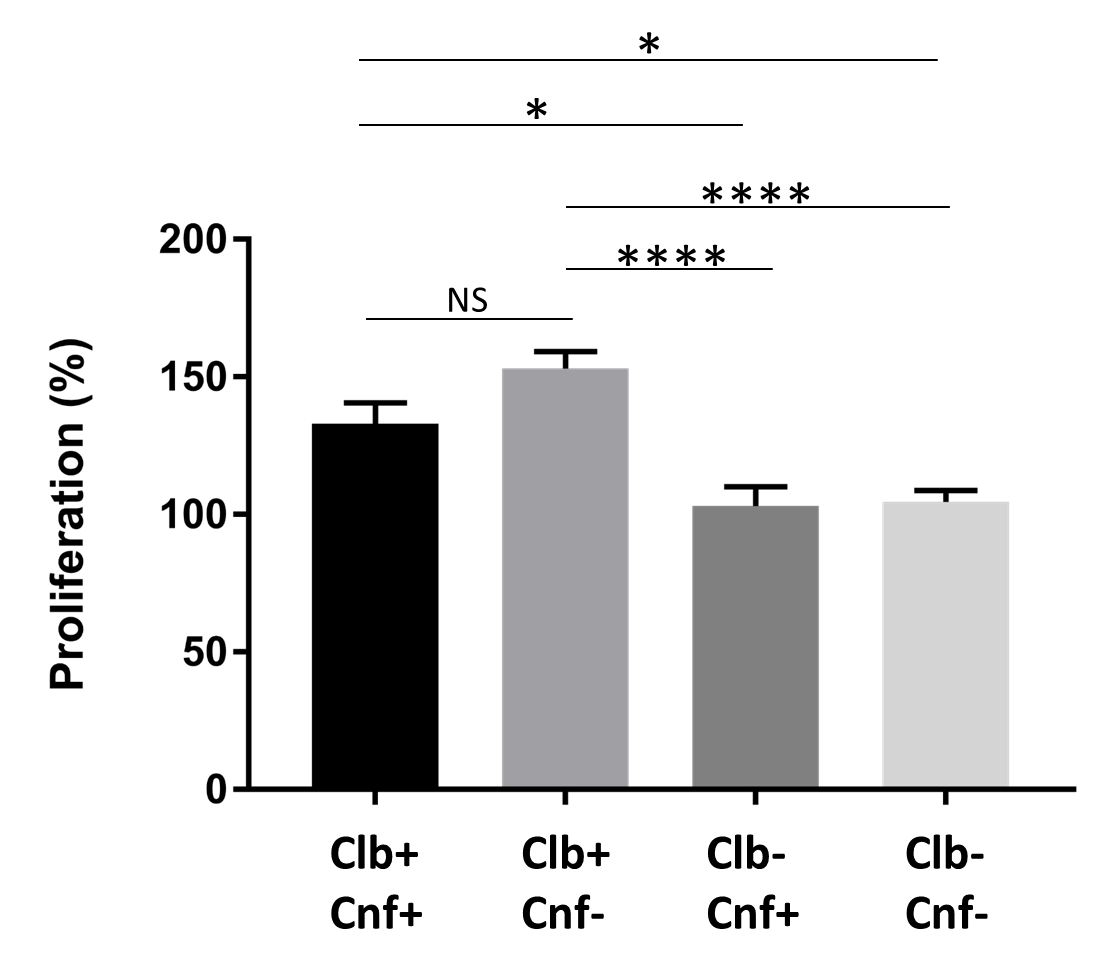


**Figure S6: The *Cnf1* gene is not involved in the pro-proliferative effect of conditioned medium derived from CoPEC-infected cells.** HT-29 cells were infected with the indicated *E. coli* 21F8 (wild-type or isogenic mutants) for 3.5 h. At day 5 post-infection, uninfected HT-29 cells were cultured in the presence of conditioned medium derived from *E. coli*-infected HT-29 cells. Cellular growth was assessed by MTT assay after 24 h of incubation. HT-29 cells cultured for 24 h in the presence of serum-free medium were set as 100% proliferation for normalization. Values represent means ± SEMs. The significance of differences was assessed by Kruskal–Wallis tests (NS, not significant; **P* < .05; *****P* ≤ .0001).


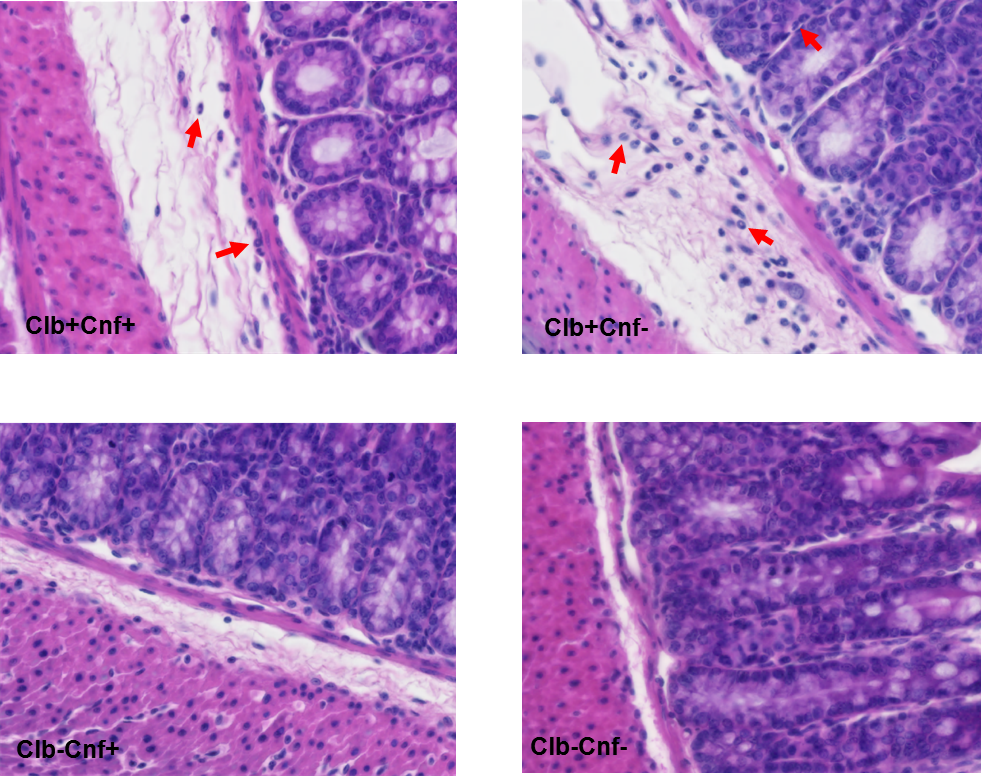


**Figure S7: The *cnf1* gene limites CoPEC-induced colonic inflammation in *Apc^Min/+^* mice.** Representative images of H&E-stained colonic sections showing submucosal oedema and inflammatory cell infiltration (scale bars: 20 µm). The arrowheads show polymorphonuclear neutrophils (with ring-shaped nuclei).


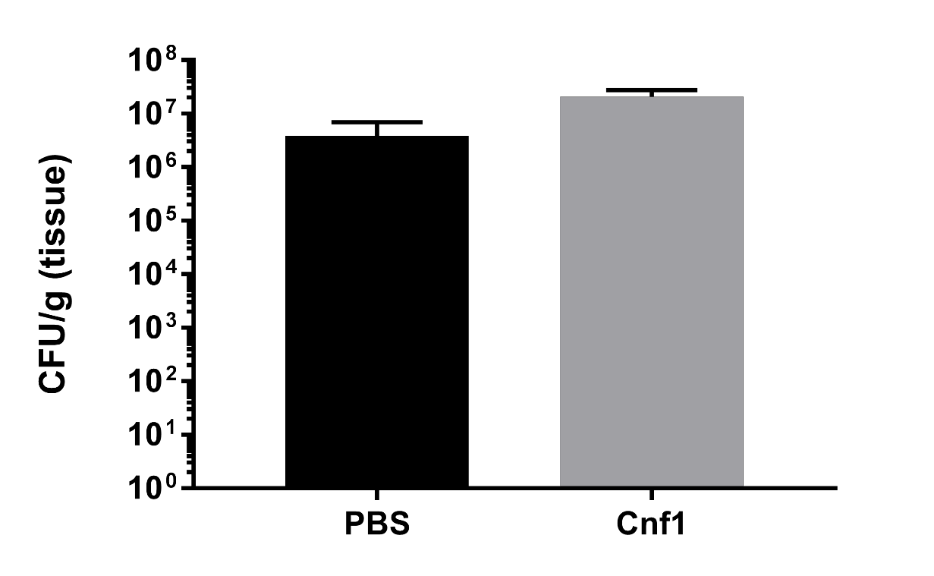


**CFU/g of colonic tissue**

NS

**Figure S8: Administration of CNF1 protein does not modify colonic colonization of Clb+Cnf- *E. coli* in *Apc^Min/+^* mice.** *Apc^Min/+^* mice received an intrarectal injection of CNF1 protein or PBS two days post-infection with the Clb+Cnf- strain and then every 7 days. The number of *E. coli* 21F8 associated with nontumoral colonic tissue at 50 days post-infection was determined. Colonization data are presented as means ± SEMs. Statistical analysis was performed using the Kruskal–Wallis test (NS, not significant).

**B**

**A**


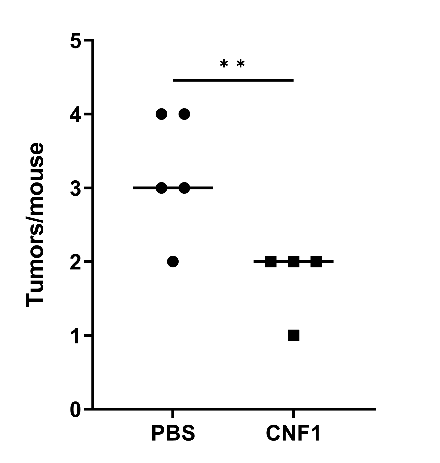

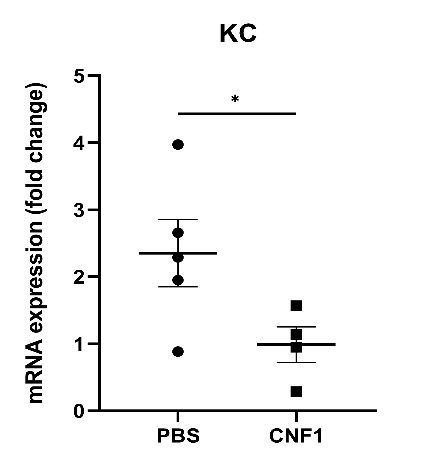

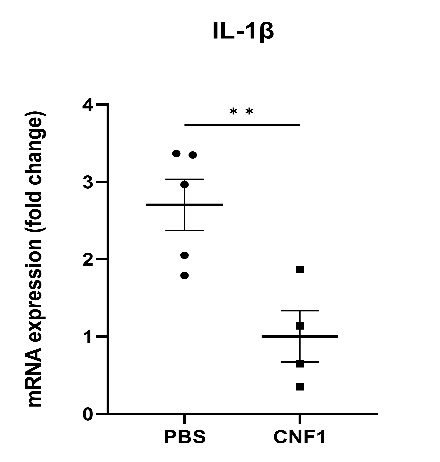

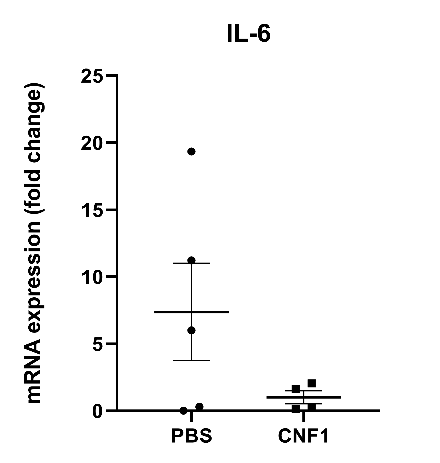

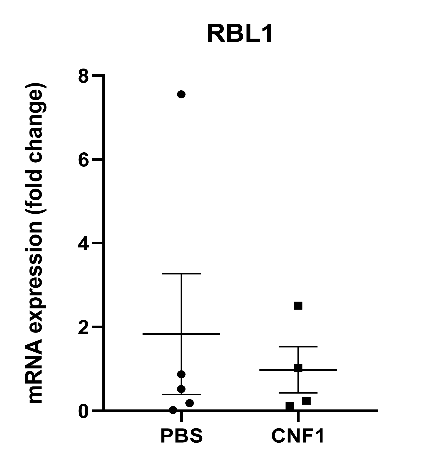

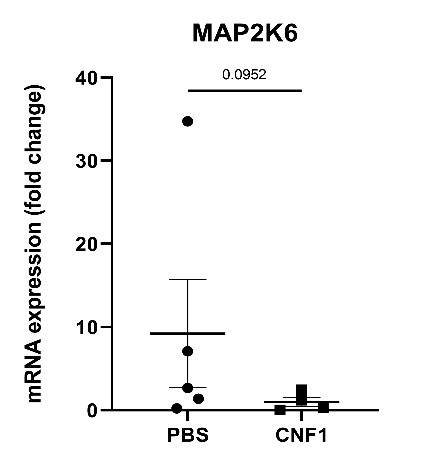


**Figure S9: In 11G5-infected *Apc^Min/+^* mice, intrarectal administration of CNF1 impedes the development of colon tumours and decreases inflammation.** *Apc^Min/+^* mice were orally administered 10^9^ colony-forming units of the *E. coli* 11G5. Two days post-infection, the mice received an intrarectal injection of 10 µg of CNF1 protein or PBS every 7 days for 7 weeks. The data points represent values for each individual mouse. (A) The number of colorectal tumours by mouse was determined using a dissecting microscope. The bars indicate median values. (B) *Kc, Il-6, Il-1β, Rbl1* and *Map2k6* mRNA relative levels in the colonic mucosa were quantified by qRT–PCR. Data are presented as means ± SEMs. Statistical comparisons were carried out by Mann-Whitney U-test.

**Table S1. Primers and plasmids used in this study**

| **Primer** | **Sequence (5’→ 3’)** | **Use** |
| --- | --- | --- |
| MIClbQaadA7 | **F:** CATTAAATCATCAAATTAAACGAATTCTATTACACAACA AGGAGTGGGACGCACTGGCATTTAATAACGCGTC  **R:** GATGATGGAACAGCCATATCTATTGCTCCTTGTATAGT TACACAACTATTTTTAATCACTTTACTTTTATC | construction of isogenic mutant |
| MIcnf1 | **F:** TCATATCCTGTTTCAATGTCCACTTCATAGTAGATGCC GCTCAGAGAACTGTAGGCTGGAGCTGCTTCG  **R:** ATGGGTAACCAATGGCAACAAAAATATCTTCTTGAGTA CAATGAGTTGGTCATATGAATATCCTCCTTAG | construction of isogenic mutant |
| TufA | **F:** GACATGGTGATGACGAAGA  **R:** GCTCTGGTTCCGGAATGTA | detection of *tufA* |
| ClbQ | **F:** GACGGCATCCACCATCGTAA  **R:** TAAATGGCGTAGCGTGCTGT | detection of *clbQ* |
| CNF1 | **F:** CGCAGTTTCAGTGATGGTGA  **R:** TCGCCAAACCAGGTATAGCA | detection of *cnf-1* |
| clbP | **F:** ACCGTGACTGATGTAAGGGC  **R:** GGCGATGAGTAACAGTTGGC | qRT–PCR amplification |
| clbM | **F:** ATTTACCAACGCCCCCAACA  **R:** TTATTCGCTGCTGACCGTGT | qRT–PCR amplification |
| clbC | **F:** TGGCAATAAGCGGGTGGAAT  **R:** TACTTACGCACAGTGGCTGG | qRT–PCR amplification |
| 36B4 | **F:** TCCAGGCTTTGGGCATCA  **R:** CTTTATCAGCTGCACATCACTCAGA | qRT–PCR amplification |
| IL-6 | **F:** TGAACAACGATGATGCACTTGCAGA  **R:** GGAGAGCATTGGAAATTGGGGTAGG | qRT–PCR amplification |
| TNF-α | **F:** CTTCCAGAACTCCAGGCGGTGC  **R:** CGGGGCAGCCTTGTCCCTTGA | qRT–PCR amplification |
| KC | **F:** TGCACCCAAACCGAAGTCAT  **R:** TTGTCAGAAGCCAGCGTTCAC | qRT–PCR amplification |
| IL1-β | **F:** TGCTGGTGTGTGACGTTCCC  **R:** TGCTGGTGTGTGACGTTCCC | qRT–PCR amplification |
| CNF-pBK | **F:** CGAGAAGCTTTTTGATCAAAATTTTTTTGAAAATACCTTC  **R:** CGACACTAGTGGATCATGGGTAACCAATGGCAAC | Clonage of *cnf* gene in pBK-CMV plasmid |
| **PlasmidRev** | **F:** GATCCACTAGTGTCGACCTGCAG  **R:** TCAAAAAGCTTCTCGAGAGTACTTC | Clonage of *cnf* gene in pBK-CMV plasmid |

| **Plasmid** | **Description** | **Use** | **Reference** |
| --- | --- | --- | --- |
| pKOBEG | pBAD cloning vector harboring a λ phage redγβα operon; chloramphenicol resistant. | Deletion of *hlyA*, *cnf1* and *clbQ* gene. | Chaveroche *et al* (2000) |
| pCPC20 | *FLP^+^*, *λ cI857^+^*, *λ*, ρ_R_ Rep^ts^, AP^R^, Cm^R^ | Excision of kanamycin cassette. | Cherepanov and Wackernagel (1995) |
| pBK-CMV-*cnf* | pBK-CMV plasmid with *cnf1-*gene, kanamycin resistant. | Clonage of *cnf1* gene. | This study |

**Table S2. Strains used in this study**

| *In vivo* strains | Description | Strain reference |
| --- | --- | --- |
| 11G5 | Clinical isolated from tumours of CRC patients harbouring *pks* island (colibactin production). | Clb+Cnf+ |
| 21F8 | Clinical isolated from tumours of CRC patients harbouring *pks* island (colibactin production) and *cnf1* (CNF1 production). | Clb+Cnf+ |
| 21F8Δ*cnf* | 21F8 strains deleted for *cnf1* gene. | Clb+Cnf- |
| 21F8Δ*clbQ* | 21F8 strains deleted for *clbQ* gene. | Clb-Cnf+ |
| 21F8Δ*cnf*Δ*clbQ* | 21F8 strains deleted for *cnf1* gene and *clbQ* gene. | Clb-Cnf- |

| *In vitro* strains | Description | Strain reference |
| --- | --- | --- |
| 21F8Δ*hlyA* | 21F8 strains deleted for *hemolysin A* gene. | Clb+Cnf+ |
| 21F8Δ*hlyA*Δ*cnf* | 21F8 strains deleted for *hemolysin A* gene and *cnf1* gene. | Clb+Cnf- |
| 21F8Δ*hlyA*Δ*clbQ* | 21F8 strains deleted for *hemolysin A* gene and *clbQ* gene. | Clb-Cnf+ |
| 21F8Δ*hlyA*Δ*cnf*Δ*clbQ* | 21F8 strains deleted for *cnf1* gene, *hemolysin A* gene and *clbQ* gene. | Clb-Cnf- |

**Supplementary results**

**The *E. coli* 21F8 *cnf1*-deleted strain was associated with alteration of the overall microbiota community structure in *Apc^Min/+^* mice.**

As both bacterial inoculation and intestinal tumour burden can affect the gut microbiota, we investigated the faecal microbiota composition by 16S rRNA gene sequencing using Illumina technology in *Apc^Min/+^* mice 50 days post-infection with wild-type 21F8 *E. coli* or its mutants. Differences in the α-diversity of the microbial communities were assessed using Shannon, InvSimpson, richness and evenness indices. There was a significant decrease in all α-diversity index values (Supplemental Fig. S10) in mice inoculated with the protumorigenic Clb+Cnf- mutant (pairwise Kruskal–Wallis tests, FDR-adjusted P value: 0.015) compared to the values in the other mice. In contrast, there was no significant difference in the α-diversity indices between the other groups (pairwise Kruskal–Wallis tests, FDR-adjusted P values: 0.130 to 0.754)

Principal coordinate analysis and hierarchical clustering based on Bray–Curtis, Jaccard and weighted UniFrac distances were performed to uncover differences in the structure of the gut microbiota of the various groups (i.e., relative abundance) at the genus taxonomic level (Supplemental Fig. S11). In agreement with the α-diversity data, the results revealed two major clusters. The first cluster included mice inoculated with protumorigenic *E. coli* Clb+Cnf-, and the second cluster, which consisted of partially overlapping subclusters, included mice inoculated with the three other *E. coli* mutants. These differences in gut microbiota composition were supported by Adonis tests, which revealed a significant difference between these groups of mice (P value of the overall test: <0.001; FDR-adjusted P values of pairwise tests: 0.014 to 0.016), with no difference in microbiota composition dispersion within the groups (Permdisp2 test, P values 0.575 to 0.748).

Networks connecting co-occurring bacteria and mutually exclusive bacteria (Supplemental Fig. S12) revealed two distinct subnetworks according to the *E. coli* genotype inoculated into mice. A well-structured subnetwork with numerous co-occurring links connecting *Bacteroides, Blautia, Anaerotruncus, Erysipelotrichaceae genus, Robinsoniella, Flavonifractor* and *Escherichia* was specifically associated with the mice inoculated with the protumorigenic *E. coli* Clb+Cnf-. This bacterial community shared dense negative correlations with the network associated with the mice inoculated with the other mutants, which were linked by a sparse network. These results were consistent with the significant changes in taxon abundance observed among the murine groups (Supplementary Fig. S13). Thus, dysbiosis was not observed in the mice inoculated with the wild-type strain 21F8, which produces both CNF1 and colibactin, whereas it was detected in the mice colonized by the protumorigenic *cnf1*-deleted isogenic mutant. Mice infected with 21F8 Clb+Cnf- strain also developed the most tumours, which was associated with an increase in the abundance of *Bacteroides* and *Anaerotruncus*, genera previously linked with CRC^2,3^. A previous metagenome-wide association study on stools from patients with advanced adenoma and CRC showed overrepresentation of *Bacteroides* and *E. coli* compared to those in stools from healthy subjects^2^. Based on a comparison of intestinal lumen samples collected from CRC patients and healthy individuals, previous studies have proposed that *Anaerotruncus* is a key phylotype that contributes to differences in the composition of the intestinal lumen microbiota in CRC patients and healthy individuals^3,4^. The congruence between microbiota composition changes and tumorigenesis mediated by colibactin-producing *E. coli* supports the hypothesis of a role for these bacteria in CRC and emphasizes the importance of the *Apc^Min/+^* murine model in investigations of the microbiota in the context of CRC.

**
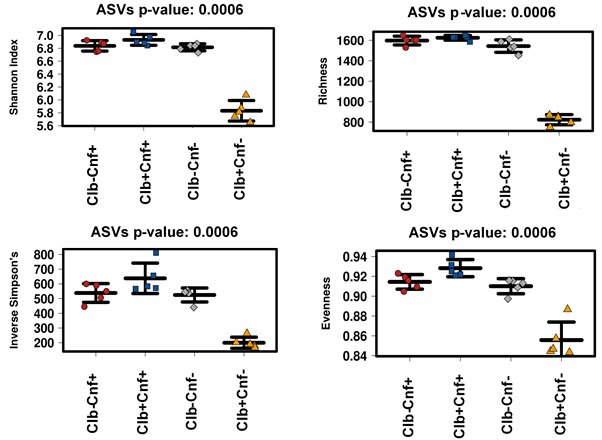
**

**Figure S10: *E. coli* Clb+Cnf- triggered a decrease in α-diversity compared to that with the isogenic *E. coli* Clb+Cnf+, Clb-Cnf+ and Clb-Cnf-.** *Apc^Min/+^* mice were orally gavaged with 10^9^ colony-forming units of isogenic *E. coli* 21F8 Clb+Cnf+, Clb+Cnf-, Clb-Cnf+ or Clb-Cnf-. Genomic DNA was extracted from faeces collected at 50 days post-infection, and the microbiota composition was inferred from 16S rRNA sequencing. The Shannon, Simpson richness and evenness α-diversity indices were calculated from counts of the 16S rRNA ASVs. The significance of differences was assessed by Kruskal–Wallis tests.

**Clb-Cnf-**


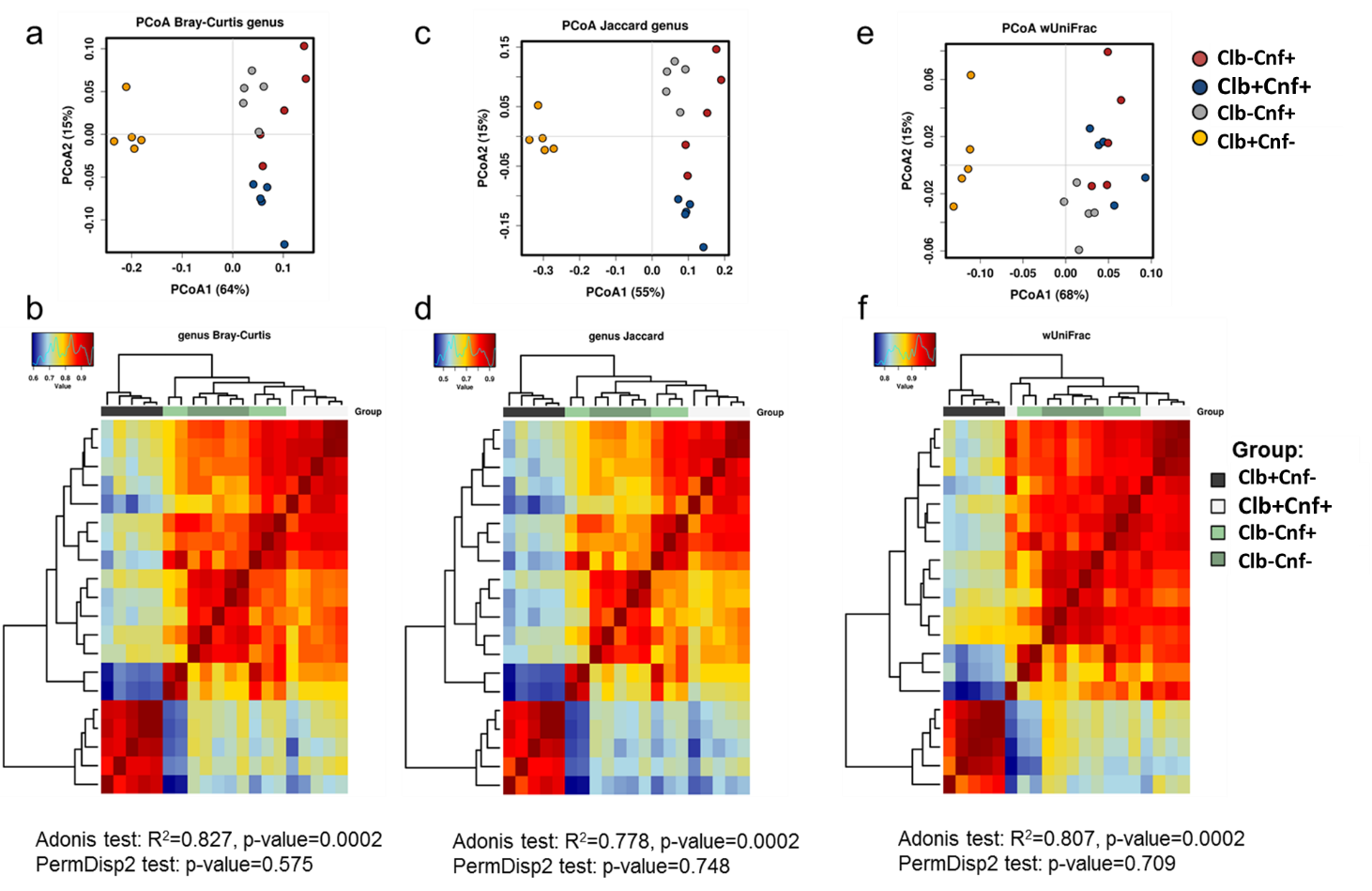


A

B

C

D

E

F

**Clb-Cnf-**

**Figure S11: The microbiota composition in mice inoculated with *E. coli* Clb+Cnf- significantly differs from that in mice infected with the wild-type strain (Clb+Cnf+) or with the isogenic mutants (Clb-Cnf+ and Clb-Cnf-).** *Apc^Min/+^* mice were orally gavaged with 10^9^ CFU of *E. coli* 21F8 Clb+Cnf+, Clb+Cnf-, Clb-Cnf+ or Clb-Cnf-. Genomic DNA was extracted from faeces collected at 50 days post-infection, and the microbiota composition was inferred from 16S rRNA sequencing. The dissimilarity in microbiota composition was assessed at the genus taxonomic level using (**A**, **B**) Bray–Curtis, (**C**, **D**) Jaccard and (**E**, **F**) weighted UniFrac indices. They are reported via principal coordinates analysis (PCoA) plots (panels **A**, **C** and **E**) and heatmaps including unsupervised hierarchical clustering (panels **B**, **D** and **F**), showing two major clusters supported by Adonis and PermDisp2 statistical tests.


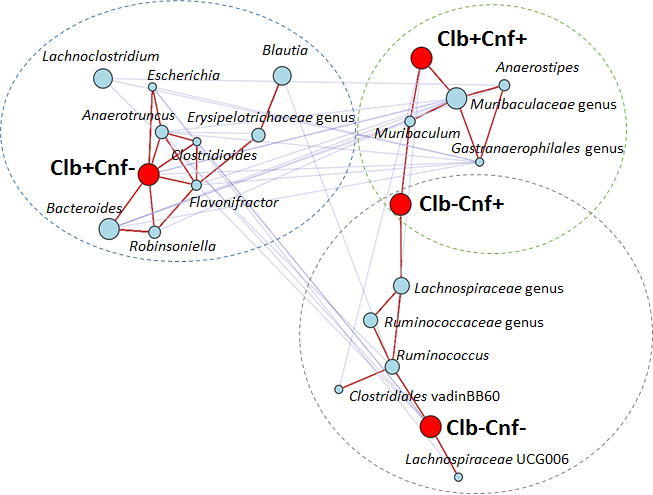


**Figure S12: Network analysis of co-occurring bacteria and mutually exclusive bacteria in the intestinal microbiota of mice inoculated with the *E. coli* 21F8 wild-type strain (Clb+Cnf+) or inoculated with the isogenic mutants Clb-Cnf+, Clb-Cnf-, and Clb+Cnf-.** Taxa and *E. coli* genotypes are represented as blue and red nodes, respectively; taxon abundance is represented as node size; and edges represent positive (dark red) and negative (blue) significant associations assessed by the ensemble method. Significant associations with *E. coli* genotypes are indicated, as well as clusters of co-occurring bacteria (dashed circles). The significance of associations was assessed by 1000-fold permutation tests, and the P values were adjusted for multiple testing by the FDR procedure.


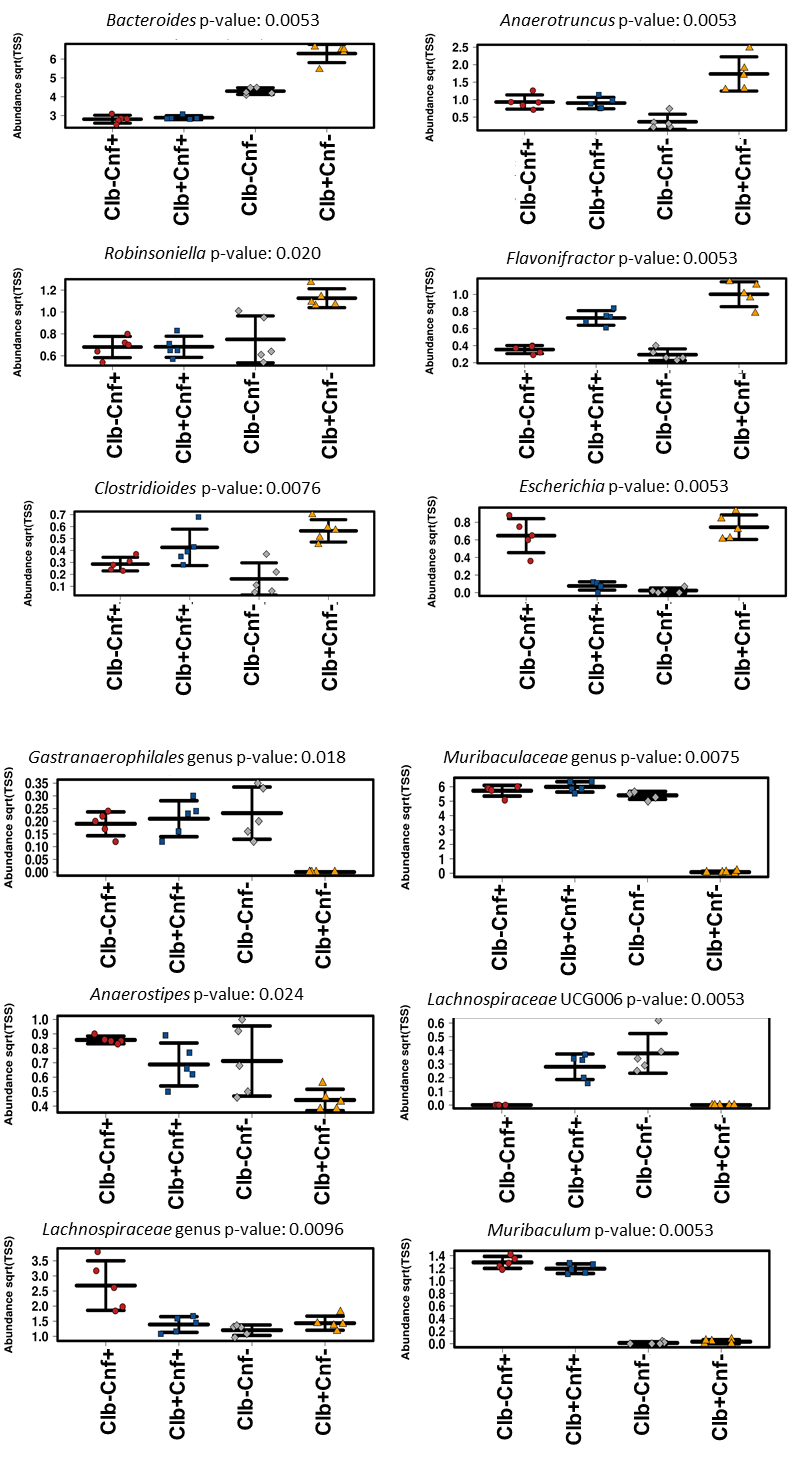


**A**

**B**

**Figure S13: Taxon abundance (spare root) in the intestinal microbiota of *Apc^Min/+^* mice inoculated with the *E. coli* 21F8 wild-type strain (Clb+Cnf+) or inoculated with the isogenic mutants Clb+Cnf-, Clb-Cnf+, and Clb-Cnf-.** Taxa over- and underrepresented in the microbiota of mice inoculated with *E. coli* Clb+Cnf- compared to those in mice inoculated with other *E. coli* mutants are reported in panels A and B, respectively. The abundances were compared by Kruskal–Wallis tests, and P values were adjusted for multiple testing by the FDR procedure.

**Supplementary Materials and Methods**

**Construction of isogenic mutants**

*E. coli* 21F8 was transformed with pKOBEG, a plasmid encoding the Red proteins that protect linear DNA from degradation in bacteria. The plasmid was maintained in bacteria at 30°C with 25 mg/L chloramphenicol and induced by 1 mM L-arabinose. The Flp recognition target-flanked cassette harbouring the kanamycin resistance cassette was generated by PCR from *E. coli* BW25141 with specific primers (Table S1) and high-fidelity Platinum Taq polymerase (Invitrogen) according to the manufacturer's instructions. The PCR products were electroporated in *E. coli* 21F8 cells previously washed with ice-cold water. The resulting 21F8Δ*cnf* and 21F8Δ*clbQ* isogenic mutants (Km^R^) were selected on Mueller-Hinton agar containing 50 mg/L kanamycin. The replacement of genes by the kanamycin resistance cassette was confirmed by PCR (Table S1). The kanamycin resistance cassette was then removed from 21F8Δ*cnf*, 21F8Δ*clbQ* and 21F8Δ*hlyA*Δ*cnf* bacteria by transient expression of the Flp recombinase from the pCP20 plasmid (Table S1), creating the sensitive-kanamycin strains used in the study. The 21F8Δ*cnf*Δ*clbQ and* 21F8Δ*hlyA*Δ*cnf*Δ*clbQ* mutant was generated with the same method (Table S2). Genes deletions and absence of additional genetic modifications were checked by analysis of whole genome sequences (2 × 15-base pairs as paired-end reads with mean sequencing depth >50x) generated by the Illumina MiSeq system (Illumina) from genomic DNA extracted with a DNeasy UltraClean Microbial Kit (Qiagen) and libraries prepared with a Nextera XT Kit (Illumina).

**Cell culture**

Human colon adenocarcinoma HT-29 cells were cultured following ATCC guidelines. HT-29 cells (~75% confluent) were infected with *E. coli* at a multiplicity of infection (MOI) of 100 for 3.5 h. After infection cells were washed using PBS and culture with regular medium supplemented with gentamycin (200 µg/mL). For cell cycle analysis (72h post-infection), cells were trypsinized for 5 min. Then cells were centrifuged for 7 min at 600 rpm and washed with PBS before fixation with 95°C ethanol at -20°C for a minimum of 30 min. After centrifugation, fixed pellet of cells was resuspended in PBS containing 15 μg/mL propidium iodide (Sigma) and 100 μg/mL RNase (ThermoFisher). Immediately after staining the cells were analysed on a LSR II Flow Cytometer ® (BD Biosciences) with at least 30,000 events and cell distribution in cell cycle phases was analyzed by FlowLogic software (Miltenyi Biotec). For γ-H2AX (24h post-infection) and p16^INK4a^ immunofluorescence staining (72 post-infection), cells were fixed with PBS with 4% formaldehyde for 10 min, permeabilized in PBS with 0.25% Triton X-100 for 10 min, and blocked with PBS with 5% foetal calf serum for 30 min. Anti-γH2AX primary antibodies (Cell Signaling, 9718) were diluted 1/400 or anti- p16^INK4a^ antibody (1/500, ab54210, Abcam) and incubated overnight at 4°C. The secondary antibody used was Alexa 488-conjugated donkey anti-rabbit (ThermoFisher, A-21206) and A488-conjugated donkey anti-mouse IgG (Jackson ImmunoResearch, 715-545-150) with a 1/400 dilution. Nuclei were stained with Hoechst 33342 (Sigma-Aldrich, B2261). Coverslips were mounted using Eukitt mounting solution (Dutscher, 045799). Images were taken using a Zeiss LSM 980 microscope. To evaluate the percentage of γH2AX- and p16^INK4a^-expressing cells, approximately 100 to 200 cells for individual wells were counted with QuPath software^5^. Thresholds for optimal detection of DAPI and 488- p16^INK4a^ signal were fixed at 10 and for 488- γH2AX, a 30-threshlod was accepted. CNF1 was purified thanks to LeMichez teams (Institute Pasteur) as described previously^6^. HT-29 infection was processed as previously with 21F8(Clb+Cnf-) strain and incubated with CNF1 purified protein (from 0.01 to 10 nM) and/or bleomycin sulfate (Sigma-Aldrich, B8416) at 200 µg/mL. The specific cytopathic effect of toxins on cultured epithelial HT-29 cells was observed with Giemsa staining. Briefly, cells were washed with PBS and fixed for 7 min at room temperature using methanol. Cells were incubated 25 min with 10-fold-diluted-Giemsa solution, washed using distilled water and observed under Zeiss LSM 980 microscope.

**Senescence-associated β-galactosidase staining (SA β-gal), MTT assay and senescence-associated secretory phenotype (SASP) analysis.**

HT-29 cells were infected as described above and grown in complete cell culture medium supplemented with 200 µg/mL gentamycin for 3 days, and SA β-gal staining was performed using a Senescence Cells Histochemical Staining Kit (Sigma-Aldrich) according to the manufacturer’s instructions. The cells were counted, and the proportion of positive (blue) cells was expressed as a percentage of the total. In order to assess pro-proliferative effect of SASP, at 5 days post-infection, cells were washed twice with PBS and further incubated for 24h in serum-free culture medium. Conditioned media derived from uninfected and infected HT-29 cells were used to culture uninfected cells for 24 h. For proliferation analysis, cells were processed with an MTT Cell Viability Assay Kit (Sigma-Aldrich) according to the manufacturer’s instructions.

An antibody array chip (Human Cytokine Array C5, RayBiotech Life) was used to evaluate 72 secreted inflammatory factors in the conditioned medium according to the manufacturer’s instructions. The conditioned medium used was a mix of 3 replicates of cell culture infected by Clb+Cnf+ or Clb+Cnf- strains. The intensity of the signal on the array membranes was quantified by densitometry using ImageJ software and normalized to positive control signals.

**Western blot**

Proteins of treated HT-29 cells were extracted with the addition of 300 μL/well of lysis buffer (20 mM Tris HCl, 150 mM NaCl, 2 mM EDTA, NP-40 1% (v/v), 0.3%, 10 nM Sodium floride (NaF), 20 mM NA_3_V0_4_, cOmplete^TM^ Protease Inhibitor Cocktail, Roche; pH 7.4). Briefly cells were washed with 1X cold PBS, 60 µl of lysis buffer was added and cells were collected with cell scrapers (Fisher), centrifuged at 10,000 rcf for 5 min at 4°C. Before to conserve supernatants to -20°C, proteins was quantified with Bio-Rad DC protein Assay. Proteins were loaded in a 15% SDS-PAGE gel for electrophoresis migration and were transferred to a nitrocellulose membrane (Trans-Blot® Transfer System, Bio-Rad). After 1 h of incubation in blocking buffer (PBS-Tween 20 0.05%, 5% BSA), membranes were blotted with the primary antibodies anti-γH2AX primary antibodies (Cell Signaling, 9718) or anti-GAPDH (1/4000, 2118, Cell Signaling) overnight at 4°C. Membranes were washed 3 times with PBS-Tween 20 0.05% and then incubated with appropriate HRP- conjugated secondary antibodies diluted in blocking buffer (1/10000) for 1 h at room temperature. Membranes were washed 3 times and proteins were detected using ECL substrate (Enhanced ChemiLuminescence, ClarityTM Western ECL Substrate, Bio-Rad). Bands intensities were quantified using Image Lab software (Bio-Rad).

**Enzyme-linked immunosorbent assays (ELISAs)**

The amount of IL-8 secreted in the supernatants from cell culture at 24 h post-infection and the amounts of KC, IL-6 and TNF-α released by mouse colonic tissues were determined by ELISA (R&D Systems) according to the manufacturer’s instructions.

**Colonization of the *Apc^Min/+^* mouse model and quantification of *E. coli* in stools and of *E. coli* associated with colonic mucosa**

Animal protocols were in accordance with French and European Economic Community guidelines (86-60, EEC) for the care of laboratory animals. Female C57BL/6j-*Apc*^Min/+^ mice (6-7 weeks of age) were randomized using the standard “= RAND( )” function in Microsoft Excel. Same litter mates were housed together in individually ventilated cages with three to five mice per cage. All mice were maintained on a regular diurnal lighting cycle (12:12 light:dark) with ad libitum access to food and water. Mice were infected with the clinical *E. coli* 11G5 or 21F8 strains or with the isogenic mutants of the 21F8 stain. To reduce the levels of endogenous gut bacteria, we administered streptomycin (2.5 g/L) for 3 days prior to oral inoculation with bacteria (∼1 × 10^9^ bacteria in PBS). To enhance *E. coli* strain colonization, 3 days after oral inoculation, we administered vancomycin (0.5 g/L) and metronidazole (0.5 g/L) for 3 days. We periodically assessed faecal bacterial colonization. Faeces were collected, crushed (Ultra-Turrax, IKA) in PBS and spread on chromID CPS Elite agar (BioMérieux) plates, which allowed the detection of *E. coli*. A random selection of 10 *E. coli*/mouse was analysed by PCR using specific primers located in the *clbQ* gene of the *pks* island and in the *cnf1* gene. Colonic tissues were treated as faeces.

**Total RNA extraction, cDNA synthesis and qRT–PCR**

Bacterial total RNA was extracted using a Direct-zol RNA MiniPrep Kit (Zymo Research) and treated with a Turbo DNA-Free Kit (Ambion) to remove any contaminating genomic DNA following the manufacturer’s instructions. DNase-treated RNA samples were purified with RNA Clean and Concentrator-25 (Zymo Research). The RNA quality was assessed by a bioanalyzer, and the RNA concentration was determined using a Qubit 2.0 fluorimeter (Thermo Fisher Scientific). mRNAs were reverse transcribed using a PrimeScript RT Reagent Kit (Takara) following the manufacturer’s instructions. qRT–PCR was performed using 2 µl of cDNA SYBR Green Master Mix (BioRad) and 100 nM specific primers (Table S1). The mouse *36B4* gene was used as an endogenous control to normalize the target gene expression. For analysis, the fold change for the target gene was calculated using the 2^−ΔΔCT^ method after normalization to the control. For qRT-PCR on bacteria culture, the *E.coli* *tufA*, *ClbP, ClbM, ClbC* genes 95 °C for 5 min, and 50 cycles of 95 °C for 15 s, 60 °C for 20 s and 72 °C for 40 s, followed by melting curve protocols with +0.5°C/s from 65°C to 95°C. For relative qRT-PCR, each gene expression was normalized to *tufA* using the 2−ΔΔCt method.

**Histological examination, scoring and immunohistochemical staining**

The colons of the animals were prepared as “Swiss rolls” and fixed with Methacarn’s solution for 24h before being embedded in paraffin. The paraffin-embedded colons were cut into 5 µm sections with a microtome, and the colonic sections were stained with haematoxylin and eosin. All tumours were histologically determined to be adenocarcinomas. The histological evaluation of the colonic sections was performed under blinded conditions by an expert pathologist using four easily identifiable pathological criteria: the extent of cellular infiltration (0-5), declining crypt architecture (crypt damage, 0-5), size and relative extent of ulceration (0-3), and absence or presence of oedema (0 and 1, respectively).

For immunohistochemical staining, the sections were deparaffinized in Histoclear*®* for 30 min, rehydrated in an ethanol gradient (100%, 95%, and 70%: 1 min each) and then incubated for 20 min at 95°C in Tris-EDTA buffer (10 mM Tris-Base, 1 mM EDTA, pH 9). Using an *in situ* hybridization and immunohistochemistry automated InSitu Pro VSi (Intavis), the sections were incubated with blocking buffer (1% bovine serum albumin in PBS) for 1 h at room temperature and then with the anti-p16^INK4a^ antibody (1/500, ab54210, Abcam) or anti-phospho-γH2AX antibody (1/560, #9718, Cell Signaling) overnight at 4 °C. After several washes with PBS, the sections were incubated for 2 h at room temperature with the corresponding biotin-SP-secondary antibody coupled with peroxidase (1/500, Jackson ImmunoResearch). Development was performed using 3,3'-diaminobenzidine (Clinisciences). The sections were then counterstained with Mayer haematoxylin (Diapath) for 10 sec, rinsed under running water, dehydrated and mounted in Eukitt mounting solution. Microscopic images were acquired using a Zeiss Axioscan Z1 Scanner (Zeiss) and analysed using ZEN 2 software. To evaluate the percentage of γH2AX- and p16-expressing cells, approximately 20 to 100 crypts were assessed in each specimen.

**Whole-genome sequencing**

Genomic DNA (gDNA) was used as input for library preparation using the Illumina Nextera XT DNA library preparation kit (Illumina, San Diego, CA, USA). Genome assembly was performed using CLC Genomics Workbench v12 (Qiagen, Les Ulis, France).

**Microbiota composition analysis**

DNA was extracted from frozen (−80 °C within 1 h) mouse faecal samples using the HMS DNA extraction protocol Q as previously described^7^. DNA samples and quality controls (ZymoBIOMICS microbial standards and a negative control) were used for sequencing the V3-V4 variable region of 16S rRNA genes using Illumina technology (2x300 paired-end reads on a MiSeq) according to Klindworth *et al* ^8^ and the manufacturer’s guidelines. Sequence data were joined with PEAR^9^ and then processed using the current recommended protocol for UPARSE^10^ (https://drive5.com/usearch/manual/uparse_pipeline.html, accessed 20th November 2020) with the UNOISE3 algorithm to generate an amplicon sequence variant (ASV) table. Singletons and any ASVs present at <0.01% relative abundance across all samples were removed before further analyses. Taxonomy assignment was performed with the SILVA database 132 (<https://www.arb-silva.de/>) and SINTAX80 classifier^11^. Subsequent analyses of diversity were performed in R using the Vegan and Phyloseq packages^12^; R package version 2.5-6, <https://CRAN.R-project.org/package=vegan>] with >10,000 sequences per sample. The data were normalized by total sum scaling and square root transformation as previously described^13^. Taxon association networks were determined according to the ensemble method as previously reported^14^. This method assesses the associations by combining Bray–Curtis dissimilarities with Pearson’s correlation and Spearman’s rho. The *P values* were computed from 1000-fold permutation tests and were adjusted for multiple testing by the FDR procedure. Significant associations (FDR-adjusted P values <0.05) are presented as edges in the resulting networks.

**References**

1. Joensen KG, Scheutz F, Lund O, Hasman H, Kaas RS, Nielsen EM, Aarestrup FM. Real-Time Whole-Genome Sequencing for Routine Typing, Surveillance, and Outbreak Detection of Verotoxigenic *Escherichia coli*. Journal of Clinical Microbiology 2014; 52:1501–10.

2. Feng Q, Liang S, Jia H, Stadlmayr A, Tang L, Lan Z, Zhang D, Xia H, Xu X, Jie Z, et al. Gut microbiome development along the colorectal adenoma–carcinoma sequence. Nature Communications 2015; 6:6528.

3. Chen W, Liu F, Ling Z, Tong X, Xiang C. Human Intestinal Lumen and Mucosa-Associated Microbiota in Patients with Colorectal Cancer. PLoS One [Internet] 2012 [cited 2021 Feb 1]; 7. Available from: https://www.ncbi.nlm.nih.gov/pmc/articles/PMC3386193/

4. Lau SKP, Woo PCY, Woo GKS, Fung AMY, Ngan AHY, Song Y, Liu C, Summanen P, Finegold SM, Yuen K. Bacteraemia caused by *Anaerotruncus colihominis* and emended description of the species. J Clin Pathol 2006; 59:748–52.

5. Bankhead P, Loughrey MB, Fernández JA, Dombrowski Y, McArt DG, Dunne PD, McQuaid S, Gray RT, Murray LJ, Coleman HG, et al. QuPath: Open source software for digital pathology image analysis. Sci Rep 2017; 7:16878.

6. Falzano L, Fiorentini C, Donneli G, Michel E, Kocks C, Cossart P, Cabanié L, Oswald E, Boquet P. Induction of phagocytic behaviour in human epithelial cells by Escherichia coli cytotoxic necrotizing factor type 1. Mol Microbiol. 1993; 6:1247–54.

7. Costea PI, Zeller G, Sunagawa S, Pelletier E, Alberti A, Levenez F, Tramontano M, Driessen M, Hercog R, Jung F-E, et al. Towards standards for human fecal sample processing in metagenomic studies. Nat Biotechnol 2017; 35:1069–76.

8. Klindworth A, Pruesse E, Schweer T, Peplies J, Quast C, Horn M, Glöckner FO. Evaluation of general 16S ribosomal RNA gene PCR primers for classical and next-generation sequencing-based diversity studies. Nucleic Acids Res 2013; 41:e1.

9. Zhang J, Kobert K, Flouri T, Stamatakis A. PEAR: a fast and accurate Illumina Paired-End reAd mergeR. Bioinformatics 2014; 30:614–20.

10. Edgar RC. UPARSE: highly accurate OTU sequences from microbial amplicon reads. Nat Methods 2013; 10:996–8.

11. Edgar RC. Accuracy of taxonomy prediction for 16S rRNA and fungal ITS sequences. PeerJ 2018; 6:e4652.

12. McMurdie PJ, Holmes S. phyloseq: An R Package for Reproducible Interactive Analysis and Graphics of Microbiome Census Data. PLOS ONE 2013; 8:e61217.

13. Paulson JN, Stine OC, Bravo HC, Pop M. Robust methods for differential abundance analysis in marker gene surveys. Nat Methods 2013; 10:1200–2.

14. Lima-Mendez G, Faust K, Henry N, Decelle J, Colin S, Carcillo F, Chaffron S, Ignacio-Espinosa JC, Roux S, Vincent F, et al. Ocean plankton. Determinants of community structure in the global plankton interactome. Science 2015; 348:1262073.
